# A Novel Mechanism for Binding of Galactose-terminated Glycans by the C-type Carbohydrate Recognition Domain in Blood Dendritic Cell Antigen 2*[Fn FN2]

**DOI:** 10.1074/jbc.M115.660613

**Published:** 2015-05-20

**Authors:** Sabine A. F. Jégouzo, Hadar Feinberg, Tabassum Dungarwalla, Kurt Drickamer, William I. Weis, Maureen E. Taylor

**Affiliations:** From the § Departments of Structural Biology and Molecular and Cellular Physiology, Stanford University School of Medicine, Stanford, California 94305 and; the ‡ Department of Life Sciences, Imperial College, London SW7 2AZ, United Kingdom

**Keywords:** carbohydrate-binding protein, crystal structure, glycobiology, glycoprotein, lectin, CD303, CLEC4C

## Abstract

Blood dendritic cell antigen 2 (BDCA-2; also designated CLEC4C or CD303) is uniquely expressed on plasmacytoid dendritic cells. Stimulation of BDCA-2 with antibodies leads to an anti-inflammatory response in these cells, but the natural ligands for the receptor are not known. The C-type carbohydrate recognition domain in the extracellular portion of BDCA-2 contains a signature motif typical of C-type animal lectins that bind mannose, glucose, or GlcNAc, yet it has been reported that BDCA-2 binds selectively to galactose-terminated, biantennary *N*-linked glycans. A combination of glycan array analysis and binding competition studies with monosaccharides and natural and synthetic oligosaccharides have been used to define the binding epitope for BDCA-2 as the trisaccharide Galβ1–3/4GlcNAcβ1–2Man. X-ray crystallography and mutagenesis studies show that mannose is ligated to the conserved Ca^2+^ in the primary binding site that is characteristic of C-type carbohydrate recognition domains, and the GlcNAc and galactose residues make additional interactions in a wide, shallow groove adjacent to the primary binding site. As predicted from these studies, BDCA-2 binds to IgG, which bears galactose-terminated glycans that are not commonly found attached to other serum glycoproteins. Thus, BDCA-2 has the potential to serve as a previously unrecognized immunoglobulin Fc receptor.

## Teaser

**Background:** BDCA-2 is an anti-inflammatory receptor uniquely expressed on plasmacytoid dendritic cells.

**Results:** Glycan arrays and x-ray structural analysis have been used to define the mechanism of sugar binding to BDCA-2.

**Conclusion:** BDCA-2 binds selectively to glycans containing the epitope Galβ1–3/4GlcNAcβ1–2Man.

**Significance:** Binding of BDCA-2 to unusual galactose-terminated glycans on IgG or other serum glycoproteins provides a potential mechanism for modulating the immune response.

## Introduction

Blood dendritic cell antigen 2 (BDCA-2)^3^ is a defining feature of human plasmacytoid dendritic cells. It was originally identified as the target for one of several monoclonal antibodies that recognize antigens that are uniquely expressed on this unusual population of circulating leukocytes (1). Plasmacytoid dendritic cells have properties often associated with classical dendritic cells, such as the ability to internalize and present antigens, but they are circulating cells that develop through a distinct lineage (2, 3). They are believed to play an important role in initial stages of the adaptive immune response and have therefore been an important target in vaccine development.

Multiple receptors that mediate internalization of antigens in antigen-presenting cells are expressed on the surface of classical dendritic cells. Several of these endocytic receptors, such as DC-SIGN and the mannose receptor, have sugar binding activity and contain Ca^2+^-dependent carbohydrate recognition domains (C-type CRDs) (4, 5). However, much less is known about C-type lectins on plasmacytoid dendritic cells. Sequence analysis reveals that BDCA-2 also contains a C-type CRD, despite the fact that it falls into a structurally distinct group of type 2 transmembrane receptors (6, 7). Although the cytoplasmic domain of the polypeptide is short and lacks obvious internalization or signaling motifs, BDCA-2 has a well defined link to signaling pathways as a result of its association with the common Fc receptor γ subunit (8, 9). Stimulation of BDCA-2 with antibodies initiates anti-inflammatory pathways through association with Syk kinase and adapter proteins such as Btk and BLNK, leading to stimulation of phospholipase C and a reduction in secretion of type 1 interferon (1, 10).

Although the sequence of BDCA-2 suggests that it contains a C-type carbohydrate recognition domain, there is conflicting evidence about how it might bind such ligands. The primary sugar-binding site in a C-type CRD is usually centered on a conserved bound Ca^2+^ that makes coordination bonds to two adjacent hydroxyl groups in the pyranose ring of a monosaccharide residue (4). A group of conserved amino acid side chains simultaneously ligate to the Ca^2+^ and form hydrogen bonds with the sugar hydroxyl groups. Some of these amino acid residues are conserved in all sugar-binding C-type CRDs, whereas others vary with the type of sugar bound. C-type CRDs containing glutamic acid and asparagine residues in the sequence EPN at the primary Ca^2+^ site bind mannose, glucose, and *N*-acetylglucosamine through equatorial 3- and 4-OH groups, whereas in CRDs that bind sugars such as galactose and *N*-acetylgalactosamine, in which the 4-OH group is axial, the corresponding sequence is QPD (11). In keeping with the presence of the EPN motif in BDCA-2, it binds to neoglycoproteins that carry mannose, GlcNAc, and glucose but not to those bearing galactose or *N*-acetylgalactosamine (12). Therefore, it is surprising that glycan array analysis indicates that BDCA-2 binds selectively to galactose-terminated biantennary glycans (13). In addition, a published structure of BDCA-2 reveals a domain-swapped structure that lacks many elements of the typical Ca^2+^-dependent binding site (14).

To clarify the apparent contradictions between the known mechanism of sugar binding to C-type CRDs and the apparent properties of BDCA-2, the ligand binding properties of the CRD have been investigated further. Binding, structural, and mutational analysis of the receptor suggest that it has a primary binding site for mannose, but that selectivity for galactose-terminated glycans is achieved through a secondary binding site. The results reconcile the existing data and help to define potential biological ligands for BDCA-2.

## Materials and Methods

### 

#### 

##### Sugars

Methyl glycosides were purchased from Sigma and Carbosynth. Egg yolk glycopeptide was purified according to the published protocol (15), except that Sephacryl S-75 and S-peptide columns (1.5 × 300 mm; GE Life Sciences) were used for gel filtration.

Biantennary oligosaccharides were prepared by digestion of the egg yolk glycopeptide (40 μmol) with peptide *N*-glycosidase (7,500 units; New England Biolabs) at 37 °C for 72 h in 100 mm Tris-Cl, pH 7.8, and was purified by chromatography on an S-peptide column run in 1% acetic acid followed by passage over an Oasis C18 column (Waters) pre-equilibrated with 5 ml of methanol and 5 ml of water. Partial desialylation was achieved by incubation in 1 ml of 40 mm HCl for 6 h at 37 °C followed by neutralization with 0.4 ml of 1 m NaHCO_3_. The oligosaccharide was purified by two passes over the S-peptide column run in 2 mm Tris. Separation of charged forms was performed by chromatography on a 1-ml column of QAE-Sephadex (A25 resin from GE Life Sciences) equilibrated in 2 mm Tris (16). Fully desialylated oligosaccharide was eluted with 6 × 1 ml of 2 mm Tris, monosialylated oligosaccharide was eluted with 6 × 1 ml of 2 mm Tris containing 20 mm NaCl, and the remaining disialylated oligosaccharide was eluted with 6 × 1 ml of 2 mm Tris containing 70 mm NaCl. Eluted pools were lyophilized and desalted on the S-peptide column run in 1% acetic acid. The separated oligosaccharides were quantified by anthrone assay and characterized by NMR and mass spectrometry.

The disaccharide GlcNAcβ1–2Man, purchased from Dextra, was extended by incubation of 25 μmol in 2 ml of 50 mm Tris-Cl, pH 7.4, containing 20 mm MgCl_2_ with 35 μmol of UDP-galactose (Sigma), 100 milliunits of galactosyltransferase from bovine milk (Sigma), and 20 milliunits of alkaline phosphatase (Sigma) for 4 h at 37 °C. The reaction was lyophilized, dissolved in 4 ml of chromatography solvent A (*n*-butanol:acetic acid:H_2_O, 3/1/1), and fractionated on a 5-ml column of silica gel. The trisaccharide product eluted between 18 and 30 ml, as determined by chromatography of aliquots on high performance thin layer silica plates that were run in solvent A and stained with orcinol. The product was further purified by gel filtration on a 15 × 300-mm S-peptide column run in water. The final product was quantified by anthrone assay and characterized by NMR and mass spectrometry (supplemental Figs. S1 and S2).

##### Affinity Resins

Mannose-Sepharose was prepared by the divinyl sulfone coupling method (17). Glycopeptide (25 mg), partially desialylated by heating at 70 °C for 30 min in 50 mm HCl and purified by gel filtration on a 15-ml Sephadex G-25 column run in 1% acetic acid, was coupled to Affi-Gel 10 (10 ml) in 6 ml of 0.1 m sodium HEPES buffer, pH 7.5, for 3 h at 4 °C.

##### Expression Plasmids

A cDNA for BDCA-2 was amplified from a human testis cDNA library (Clontech/Takara) by PCR (Advantage 2 polymerase mix; Takara), cloned into the pCR2.1-TOPO vector (Invitrogen), and sequenced using an Applied Biosystems 310 genetic analyzer. The portion of the cDNA encoding the CRD was reamplified with forward primers designed to provide an initiation sequence in the pT5T expression vector (18). In addition to the natural termination codon, a version with nucleotides encoding the biotinylation sequence Gly-Leu-Asn-Asp-Ile-Phe-Glu-Ala-Gln-Lys-Ile-Glu-Trp-His-Glu (19) at the 3′ end was created using an alternative reverse primer. Mutagenesis was performed by two-step PCR (20) using the original cDNA clone as template. Expression was performed in *Escherichia coli* strain BL21(DE3), which was co-transformed with pBirA plasmid encoding biotin ligase (19) for expression of biotin-tagged proteins.

##### Protein Purification and Analysis

Expression of the wild type CRD from human BDCA-2 following published protocols for other C-type CRDs resulted in inclusion bodies that were isolated as described (21). Inclusion bodies from 2 liters of bacterial culture were dissolved in 30 ml of 6 m guanidine HCl containing 100 mm Tris-Cl (pH 7.8) and incubated in the presence of 0.01% (v/v) 2-mercaptoethanol for 30 min at 4 °C. Following centrifugation for 30 min at 100,000 × *g* in a Beckman Ti70.1 rotor, the supernatant was diluted dropwise into 120 ml of 0.5 m NaCl, 25 mm Tris-Cl, pH 7.8, and 25 mm CaCl_2_ at 4 °C, followed by dialysis against 2 changes of 2 liters of the same buffer. Insoluble material was removed by centrifugation for 30 min at 50,000 × *g* in a Beckman JA20 rotor, and the supernatant was applied to a 5-ml column of glycopeptide-agarose. After rinsing with 12 ml of 150 mm NaCl, 25 mm Tris-Cl, pH 7.8, and 25 mm CaCl_2_, the bound protein was eluted with 10 × 1-ml aliquots of 150 mm NaCl, 25 mm Tris-Cl, pH 7.8, and 2.5 mm EDTA. Fractions containing the CRD were identified by analyzing aliquots on SDS-polyacrylamide gels, with protein detected by staining with Coomassie Blue.

Mutant forms of the CRD were expressed in the same way, but following initial dialysis against the renaturation buffer, the proteins from 4–6 liters of culture were further dialyzed against two changes of 2 liters of H_2_O and lyophilized. The lyophilized proteins were taken up in 6 ml of 150 mm NaCl, 25 mm Tris-Cl, pH 7.8, and 25 mm CaCl_2_ and centrifuged at 100,000 × *g* in a Beckman TLA100.4 rotor for 30 min at 4 °C. The supernatant was applied to a 10-ml column of mannose-Sepharose, which was washed five times with 2-ml aliquots of 150 mm NaCl, 25 mm Tris-Cl, pH 7.8, and 25 mm CaCl_2_ and eluted with three 2-ml aliquots and eight 1-ml aliquots of 50 mm NaCl, 25 mm Tris-Cl, pH 7.8, and 2.5 mm EDTA.

Gel filtration was performed on a 1 × 30-cm Superdex 200 column (GE Healthcare) eluted with 10 mm Tris-Cl (pH 7.8), 100 mm NaCl, and 2.5 mm EDTA at a flow rate of 0.5 ml/min, with absorbance monitored at 280 nm. Gel electrophoresis was performed on SDS-polyacrylamide gels containing 17.5% (w/v) acrylamide.

##### Glycan Binding Assays

Biotinylated protein was incubated overnight with Alexa 488-labeled streptavidin (Invitrogen) at a ratio of ∼2 mol of CRD to 1 mol of streptavidin subunit. The mixture was applied to a 1-ml column of mannose-Sepharose, which was washed with loading buffer, and the complex was eluted with 0.5-ml aliquots of elution buffer. The protein was tested against version 5.1 of the glycan array of the Consortium for Functional Glycomics using the standard protocol.

Competition binding assays were performed as previously described for mincle (22). ^125^I-Man-BSA and ^125^I-IgG reporter ligands were prepared by radioiodination (23) of Man_31_-BSA (E-Y Laboratories) and human IgG (Sigma).

##### Crystallization, Data Collection, and Structure Determination

Crystals of human BDCA-2 complexed with α-methyl mannoside were grown by hanging drop vapor diffusion at 22 °C using a mixture of 0.13:0.13 μl of protein:reservoir solution in the drop, with the protein solution comprising 5 mg/ml CRD from BDCA-2, 5 mm CaCl_2_, 10 mm Tris-Cl, pH 8.0, 25 mm NaCl, and 50 mm α-methyl mannoside. The reservoir solution contained 0.2 m MgCl_2_ and 20% polyethylene glycol 3.35 K. Crystals were dipped in a freezing solution containing 30% polyethylene glycol 3.35 K, 0.2 m MgCl_2_, 5 mm CaCl_2_, 10 mm Tris, pH 8.0, 25 mm NaCl, and 50 mm α-methyl mannoside, before being frozen in liquid nitrogen for data collection. Diffraction data were measured at 100 K on Beamline 23-ID-D at the Advanced Photon Source of Argonne National Laboratory.

Crystals of human BDCA-2 complexed with Galβ1-4GlcNAcβ1–2Man were grown using a mixture of 0.2:0.1 μl of protein:reservoir solution, at 22 °C, from a protein solution comprising 6.2 mg/ml BDCA-2, 5 mm CaCl_2_, 10 mm Tris-Cl, pH 8.0, 25 mm NaCl, and 20 mm Galβ1–4GlcNAcβ1-2Man. The reservoir solution contained 0.2 NH_4_Cl and 20% polyethylene glycol 3.35 K. Crystals were dipped in oil (Lancaster PFO-XR75) before being frozen in liquid nitrogen for data collection. Diffraction data were measured at 100 K on Beamline 12-2 at Stanford Synchrotron Radiation Laboratory. All diffraction data were integrated with XDS (24) and scaled with AIMLESS (25). The statistics are summarized in Table 1.

**TABLE 1:** **Crystallographic data statistics**

Data	BDCA-2 with α-methyl mannoside	BDCA-2 with Galβ1–4GlcNAcβ1–2Man
Symmetry	P22_1_2_1_	P22_1_2_1_
Wavelength (Å)	1.03319	0.97950
Unit cell lengths (Å)	*a* = 37.73, *b* = 69.58, *c* = 117.51	*a* = 37.56, *b* = 67.64, *c* = 118.14
Resolution Å (last shell)	28.88–1.65 (1.69–1.65)	37.56–2.90 (3.07–2.90)
*R*_sym_*^a^*	5.4 (54.6)	14.6 (47.2)
Mn(I) half-set correlation CC(1/2)	0.999 (0.749)	0.985 (0.823)
Mean (*I*/σ(*I*))	17.5 (2.5)	8.7 (4.3)
% complete	99.8 (96.5)	99.2 (97.4)
Number of unique reflections	38138	7094
Average multiplicity	6.4 (4.9)	5.3 (5.2)

*^a^ R*_sym_ = 100 × Σ_h_Σ_i_ (|*I*_i_(*h*) − <*I*(*h*)>|)/Σ_h_Σ_i_*I*_i_(*h*), where *I*_i_(*h*) = observed intensity, and <*I*(*h*)> = mean intensity obtained from multiple measurements.

The high resolution structure of BDCA-2 complexed with α-methyl mannoside was solved by molecular replacement, using the program Phaser (26). The model used for molecular replacement was prepared from the coordinates for the CRD of cow mincle (Protein Data Bank entry 4ZRV), with three Ca^2+^ molecules and no carbohydrate or water molecules. The molecular replacement solution confirmed that the space group was P22_1_2_1_, with two monomers in the asymmetric unit. The lower resolution structure of BDCA-2 complexed with Galβ1-4GlcNAcβ1–2Man was solved using the partially refined high resolution structure of BDCA-2 complexed with α-methyl mannoside. Difference Fourier maps clearly showed Galβ1-4GlcNAcβ1–2Man in both monomers. Model building and refinement were performed with Coot (27) and PHENIX (28). Refinement included individual positional and isotropic temperature factor refinement and occupancy refinement for residues with alternate conformations. Refinement statistics are shown in Table 2.

**TABLE 2:** **Crystallographic refinement statistics**

Data	BDCA-2 with α-methyl mannoside	BDCA-2 with Galβ1–4GlcNAcβ1–2Man
Number of reflections used for refinement	38083	7062
Reflections marked for *R*_free_	1,905	354
*R*_free_*^a^*	21.4	26.3
*R*_cryst_*^a^*	17.3	17.8
**B factors (Å^2^)**		
Average	26.0	32.1
Protein	24.7	32.1
Ca^2+^	21.6	22.0
Sugars	26.5	31.8
Waters	34.0	
Bond length root mean square deviation (Å)	0.011	0.013
Angle root mean square deviation (°)	1.03	1.18
Ramachandran plot: (% in each region)*^b^* (preferred/allowed/outliers)	93.5/5.4/1.1	89.3/8.3/2.4

*^a^ R* and *R*_free_ = 100 × Σ_h_|*F*_o_(*h*) − *F*_c_(*h*)|/Σ_h_*F*_o_(*h*), where *F*_o_(*h*) = observed structure factor amplitude, and *F*_c_(*h*) = calculated structure factor amplitude for the working and test sets, respectively.

*^b^* As defined in Coot.

## Results

### 

#### 

##### Expression of the CRD from BDCA-2

**FIGURE 1.**
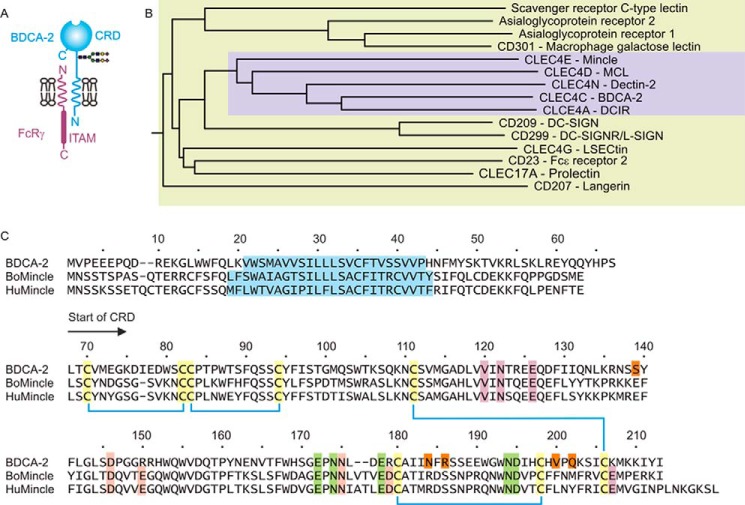
**Sequence of human BDCA-2.** *A*, summary of the organization of BDCA-2 and other type 2 transmembrane receptors containing C-type CRDs. *B*, dendrogram showing the relationships between the CRD portions of this group of C-type lectins. The subgroup containing mincle and BDCA-2 is highlighted in the *violet box. C*, sequence of BDCA-2 compared with sequences of cow and human mincle. The N terminus of the CRD is denoted by the *arrow*. Disulfide bonds are indicated by *blue lines* between conserved cysteine residues, which are highlighted in *yellow*. Amino acid residues that create the conserved Ca^2+^-binding site are indicated in *green*, and residues in mincle that create the two accessory Ca^2+^-binding sites in mincle are highlighted in *violet* and *pink*. Residues that form part of the extended sugar-binding site of BDCA-2 are highlighted with *orange*. The putative transmembrane domains are shown in *blue*.

Comparison of both the overall structural organization of BDCA-2 and the sequence of the carbohydrate recognition domain place it in a subgroup of the family of type II transmembrane receptors that includes the macrophage-inducible C-type lectin (mincle), the macrophage C-type lectin, dendritic cell C-type lectin (dectin-2), and the dendritic cell immunoreceptor (DCIR) (Fig. 1, *A* and *B*) (7). Comparison of the sequences of the CRDs of mincle and BDCA-2 (Fig. 1*C*) reveals conservation of structural framework residues including disulfide bonds and the features associated with the characteristic sugar-binding site in C-type CRDs, including five amino acids that form the conserved Ca^2+^-binding site.

**FIGURE 2.**
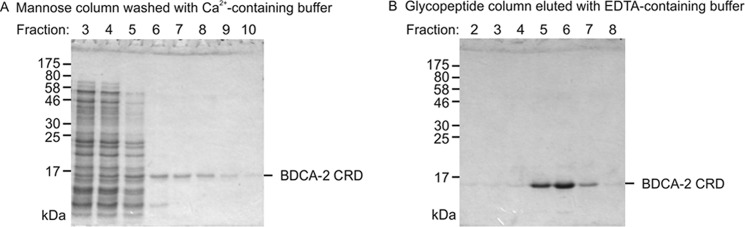
**Purification of the CRD from BDCA-2 by affinity chromatography.** *A*, the CRD was renatured from inclusion bodies by dialysis against Ca^2+^-containing buffer followed by dialysis against water and lyophilization to obtain a concentrated sample that was applied to a 10-ml column of mannose-Sepharose. After application of the sample, the column was eluted with 150 mm NaCl, 25 mm Tris-Cl, pH 7.8, 25 mm CaCl_2_. Fractions of 2 ml were collected. *B*, following renaturation by dialysis, the CRD was applied directly to a 5-ml column of desialylated egg yolk glycopeptide immobilized on agarose. The column was rinsed with 25 ml of 150 mm NaCl, 25 mm Tris-Cl, pH 7.8, 25 mm CaCl_2_, and protein was eluted with 150 mm NaCl, 25 mm Tris-Cl, pH 7.8, 2.5 mm EDTA in 1-ml fractions. In both cases, aliquots (15 μl) from fractions were analyzed on SDS-polyacrylamide gels that were stained with Coomassie Blue. The expected molecular weight of the BDCA-2 CRD is 17,100.

Following expression of the CRD from BDCA-2 in *E. coli* as inclusion bodies, several protocols for renaturation were investigated, and attempts were made to purify the CRD on high density mannose-Sepharose resin, by analogy to protocols used for mincle and other C-type lectins. When renatured protein was applied to an extended affinity column in a small sample volume, retardation of a band corresponding to the CRD from BDCA-2 was observed (Fig. 2*A*). Although the protein did not stick tightly to the column and washed through in the presence of Ca^2+^, the results provide evidence that BDCA-2 has at least weak mannose binding activity.

Based on the previous finding that a galactose-terminated biantennary glycan is a ligand for BDCA-2 (13), an alternative affinity resin was created by immobilizing a desialylated preparation of glycopeptide bearing a biantennary glycan, isolated from chicken egg yolks. With this oligosaccharide resin, the expressed CRD of BDCA-2 bound tightly in the presence of Ca^2+^ and could be eluted with EDTA (Fig. 2*B*). This approach provided a rapid method for isolation of natively folded protein and confirmed that BDCA-2 binds to the desialylated oligosaccharide in a Ca^2+^-dependent manner. In addition to the untagged CRD, a version in which a biotinylation tag was attached to the C terminus was also prepared by the same procedures.

##### Analysis of Sugar Binding

**FIGURE 3.**
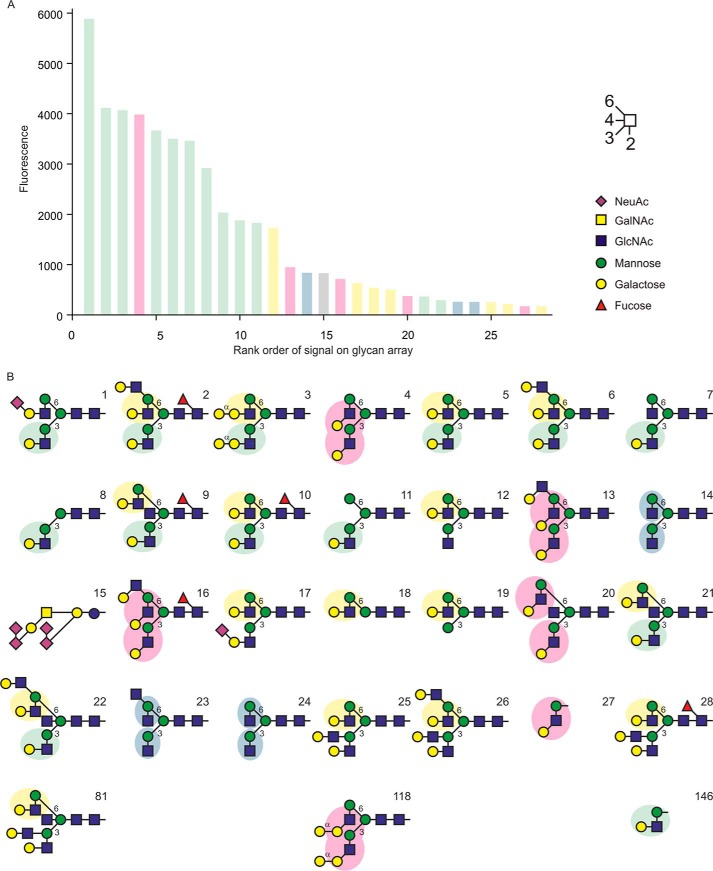
**Glycan array analysis of ligand binding to BDCA-2.** A complex of biotin-tagged CRD from BDCA-2 with Alexa 488-labeled streptavidin was used to screen synthetic glycan array version 5.1 at the Consortium for Functional Glycomics at a concentration of 100 μg/ml. *A*, results are arranged in rank order based on decreasing signal. *B*, structures corresponding to the 28 top-ranked glycans, plus three additional glycans with potential binding epitopes, are shown in symbol representation, in which the linkages from NeuAc are α2 and all other linkages are β1. The mannose α1–3 and α1–6 branch point linkages indicated with *3* and *6. Bars* in *A* are color-coded based on the presence of the shaded binding epitopes in *B*: *blue* for uncapped GlcNAcβ1–2Man, *yellow* and *green* for Galβ1–4GlcNAcβ1–2Man on the 1–3 and 1–6 arms, respectively, and *pink* for Galβ1–3GlcNAcβ1–2Man. After glycan 28, with a signal of 171, the signal drops to 109 for glycan 29, and the average signal for the remaining glycans is 17. Complete glycan array results are provided in supplemental Table S1.

The biotin tag attached to the C terminus of the CRD was used to generate tetrameric complexes of CRD with fluorescently labeled streptavidin for screening of the most recent version of the glycan array at the Consortium for Functional Glycomics, which has been expanded to 610 glycans (29). Screening of the expanded array confirmed binding to biantennary glycans with terminal galactose residues, as previously observed with versions of the array containing 264 and 320 glycans (13), and revealed a number of additional ligands. However, there were still a limited number of strong signals (Fig. 3). The 13 highest signals were obtained for glycans bearing terminal Galβ1–4GlcNAcβ1–2Man or Galβ1–3GlcNAcβ1–2Man epitopes. All but two of the glycans containing such epitopes appear in the top 28 signals. However, the signals are consistently lower when the epitope is displayed on the 1–3 branch of bi- or triantennary glycans. Glycans bearing just a trisaccharide epitope bind relatively weakly, but these trisaccharides are displayed on a relatively short two-carbon linker that may restrict access to the ligand-binding site. These results suggest that the primary ligands for BDCA-2 are a very specific subset of galactose-terminated glycans. Three of the remaining glycan ligands that lack terminal galactose residues contain terminal GlcNAcβ1–2Man epitopes on two branches, whereas seven other glycans that contain one or more copies of this terminal epitope, without galactose, rank lower on the array (glycans ranked 48, 49, 65, 100, 110, 305, and 361), suggesting that this epitope binds more weakly. The only glycan that does not fit into one of these patterns is the 15th ranked signal. Previous versions of the array had no tri- or tetra-antennary glycans, which is why these ligands were not previously detected.

Inhibition of binding is observed as a result of the following modifications of galactose: fucose at the 2 position; NeuAc, galactose, GlcNAc, or GalNAc at the 3 position; or NeuAc at the 6 position. In addition, fucose attached to the 3-OH group of GlcNAc in the Galβ1–4GlcNAcβ1–2Man epitope or to the 4-OH group of the Galβ1–3GlcNAcβ1–2Man epitope also inhibits binding. As a result, many common blood group antigens, include the ABO oligosaccharides; the Lewis x, y, a, and b epitopes; the GalαGal structure; and poly *N*-acetyllactosamine chains do not bind. Addition of sugars at the 4 position of the galactose residue is tolerated, because a glycan bearing Galα1–4Gal on two branches is also bound. However, this epitope is not commonly found in humans or other mammals (30).

**FIGURE 4.**
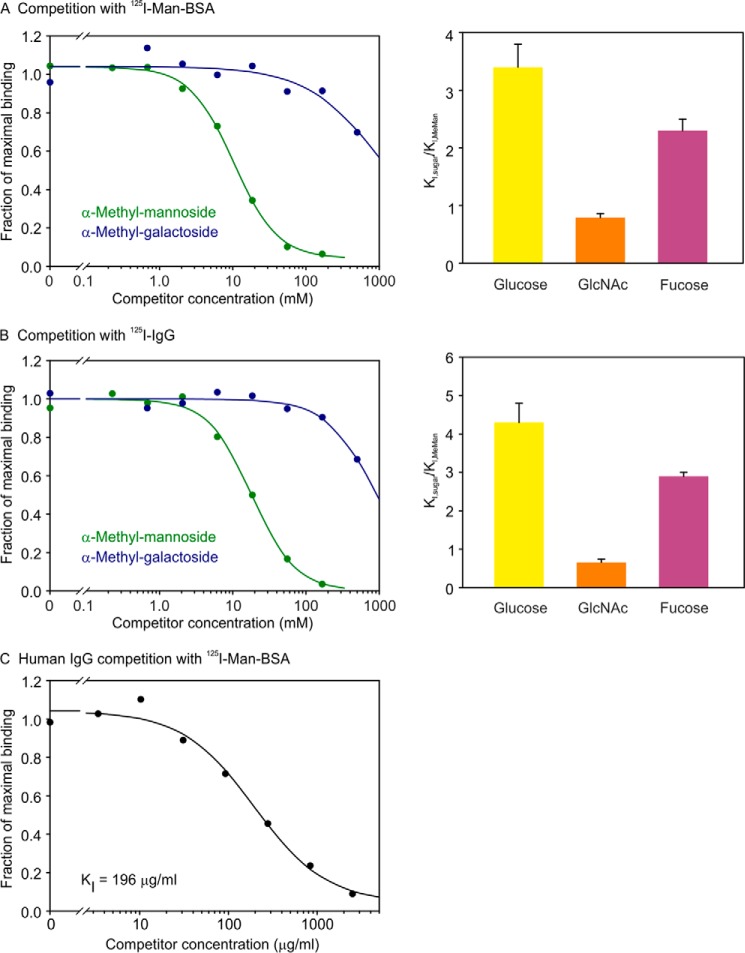
**Competition results to define primary sugar-binding site in BDCA-2.** Binding was performed with biotin-tagged CRD from BDCA-2 bound to streptavidin-coated wells. *A* and *B*, binding of ^125^I-Man-BSA or ^125^I-IgG was measured in the presence of competing α-methyl glycosides. *C*, binding of 125I-Man-BSA was measured in the presence of competing IgG. Half-maximal inhibition values (*K_I_*) were obtained by nonlinear least squares fitting of the data. The values of *K_I_* relative to the *K_I_* for α-methyl mannoside in bar graphs represent the means ± standard deviations for two to four independent assays.

Competition binding studies were performed to investigate the apparent contradiction between preferential binding to galactose-terminated glycans seen on the glycan array and the predicted specificity of the primary binding site for mannose-type ligands. The biotin tag provided an efficient means of immobilizing the CRD in streptavidin-coated wells for binding assays. Based on the earlier studies with neoglycoprotein ligands (31) and the column results demonstrating weak interactions with mannose, ^125^I-labeled Man-BSA was used as a reporter ligand so that relatively low affinity interactions with monosaccharides could be explored by competition. In all cases, methyl glycosides were used in competition studies to avoid potentially confounding interactions involving the free 1-OH group (32). Comparison of mannose and galactose as competing ligands confirmed the affinity chromatography results, because the *K_I_* for mannose was found to be at least 100-fold lower than the *K_I_* for galactose (Fig. 4*A*). As with other C-type CRDs that bind mannose, sugars that share the equatorial arrangement of 3- and 4-OH groups, including glucose and GlcNAc, as well as l-fucose, also compete for binding.

To eliminate the possibility that there are two separate sugar-binding sites in the CRD, the competition experiments were also performed using ^125^I-labeled human IgG as the reporter ligand. The glycans attached to the Fc portion of IgG are all of the complex type, predominantly terminating in galactose (33). Based on the known glycan composition of IgG and the preference for glycans seen on the glycan array, this glycoprotein would be expected to bind through the site that binds galactose-terminated glycans. Nevertheless, the binding competition results revealed the same preference for mannose over galactose as competing ligands, with exactly the same relative binding specificity for other monosaccharide ligands (Fig. 4*B*). These results argue strongly for the presence of a single binding site, a conclusion supported by the ability of the IgG to compete for binding of ^125^I-Man-BSA (Fig. 4*C*).

One explanation for the glycan array and monosaccharide competition data would be that in the most common binding epitope Galβ1–4GlcNAcβ1–2Man, the mannose binds in the primary binding site, and the galactose occupies an adjacent secondary binding site. This result would be consistent with the finding that only complex glycans in which one of the core mannose residues has exposed 3- and 4-OH groups are ligands for BDCA-2.

##### Characterization of a Primary Sugar-binding Site in BDCA-2

**FIGURE 5.**
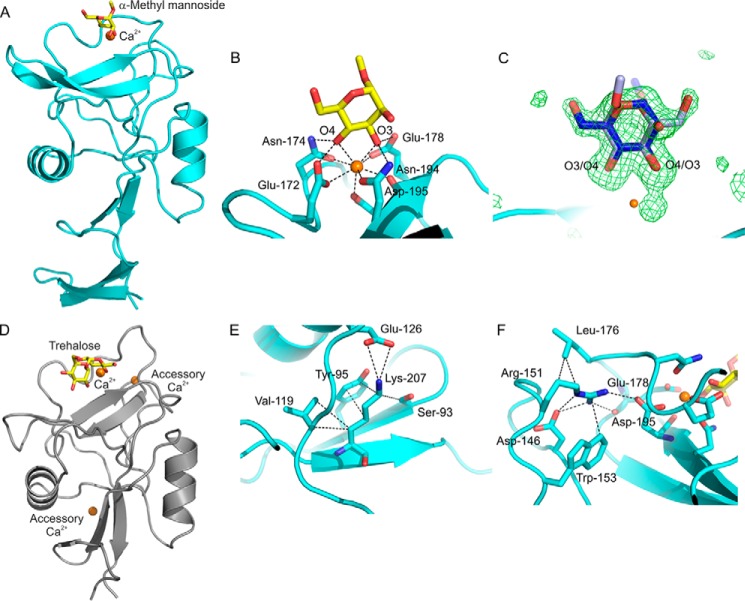
**Structural analysis of the primary sugar-binding site in BDCA-2.** *A*, overall structure of BDCA-2 with bound α-methyl mannoside. *B*, Ca^2+^ and α-methyl mannoside in the primary site of BDCA-2 monomer B. *C*, α-methyl mannoside in the primary site of monomer A. *F*_o_ − *F*_c_ map at 2.5 σ showing two conformations of the sugar related by a ∼180° rotation that switches the positions of O_3_ and O_4_. The occupancies of the conformations indicated in *dark blue*:*light blue* are 0.54:0.46, with the *dark blue* conformation corresponding to what is seen in monomer B. *D*, structure of the CRD from cow mincle (Protein Data Bank entry 4ZRV), with trehalose bound at the conserved Ca^2+^ and two accessory Ca^2+^ highlighted. This structure (H. Feinberg, S. A. F. Jégouzo, M. E. Taylor, K. Drickamer, and W. I. Weis, unpublished observations), which was used as the search model for the molecular replacement solution, is similar to Protein Data Bank entry 4KZV, but with a third Ca^2+^ replacing the bound Na^+^, in a position analogous to that seen in mannose-binding protein and other C-type CRDs (4). *E* and *F*, arrangement of amino acid residues in BDCA-2 in the positions at which two accessory Ca^2+^ are bound to the CRD of mincle. Ca^2+^ is shown in *orange*, oxygen atoms in *red*, nitrogen atoms are *dark blue*, and carbon atoms of the sugars are *yellow*.

To investigate the nature of the primary sugar-binding site in BDCA-2, crystallization of the CRD was attempted in the presence of Ca^2+^ and α-methyl mannoside to allow binding of the monosaccharide ligand. Crystals that diffracted to 1.65 Å were obtained starting from a solution containing 5 mm CaCl_2_ and 50 mm α-methyl mannoside. The structure was solved by molecular replacement, using a search model consisting of the CRD from cow mincle (Protein Data Bank entry 4ZRV), with sugar ligand removed (Fig. 5*A*). There are two copies of the molecule in the asymmetric unit. The overall fold of the main portion of the CRD is similar to mincle and other C-type CRDs, with the N-terminal extension forming an additional pair of β strands linked by a disulfide bond. The electron density maps indicated the presence of only one Ca^2+^ per monomer, bound by the five conserved residues noted in Fig. 1*C*, to create the primary sugar-binding site (Fig. 5, *B* and *C*). α-Methyl mannoside is bound to this Ca^2+^ in both copies. In copy A, sugar is bound in two alternate orientations, with roughly the same occupancies, related by a 180° rotation that interchanges the positions of the 3- and 4-OH groups. These hydroxyl groups interact with the Ca^2+^ and its amino acid ligands by the network of coordination and hydrogen bonds that are characteristic of C-type CRDs (32).

**FIGURE 6.**
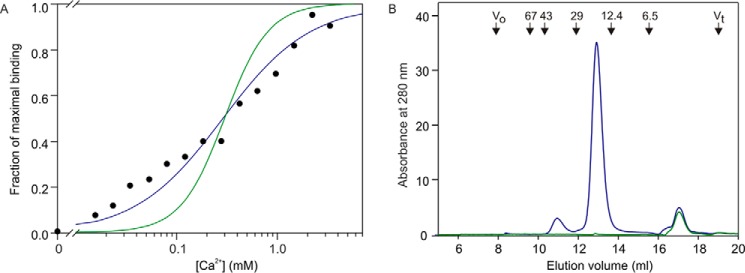
**Ca^2+^ dependence and gel filtration analysis of the CRD from BDCA-2.** *A*, Ca^2+^ dependence of ^125^I-Man-BSA binding to BDCA-2. Binding of ^125^I-mannose-BSA to the biotin-tagged CRDs immobilized in streptavidin-coated wells was quantified. After binding of ligand in 150 mm NaCl and 25 mm Tris-Cl, pH 7.8, in the presence of various concentrations of Ca^2+^, wells were washed with buffer containing 150 mm NaCl, 25 mm Tris-Cl, pH 7.8, and 25 mm CaCl_2_. The experimental data, shown as *black circles*, were fitted to first and second order binding equations, shown respectively as *blue* and *green lines*, using a nonlinear least squares fitting program. *B*, gel filtration analysis of the CRD from BDCA-2 on a 7.5 × 300-mm column of Sephacryl S75 eluted with 100 mm NaCl, 10 mm Tris-Cl, pH 7.8, and 2.5 mm EDTA at a flow rate of 0.5 ml/min. *Blue trace* is with protein, and *green trace* is a mock sample without protein, showing that the peak eluting at 17 ml results from the presence of Ca^2+^-EDTA complex in the sample. Positions of molecular weight standards are shown at the *top*.

Although the search model contained three Ca^2+^, a striking difference between the crystal structure of BDCA-2 and the structure of mincle is the absence of the two accessory Ca^2+^-binding sites (Fig. 5*D*), one near the conserved site and one on the other side of the CRD, in BDCA-2. Notably, several of the acidic amino acid side chains that interact with these two additional Ca^2+^ in mincle have been substituted by basic amino acids (Fig. 1*C*). In the structure, the side chains of two of these basic residues interact directly with the remaining acidic amino acid side chains (Fig. 5, *E* and *F*). Measurement of the effect of Ca^2+^ concentration on ^125^I-Man-BSA binding to BDCA-2 confirmed that binding is Ca^2+^-dependent, but rather than being second order with respect to Ca^2+^ as was found for mincle (22), the binding is best fitted by first order dependence, consistent with the presence of the single Ca^2+^ site observed in the crystal structure (Fig. 6*A*).

Gel filtration analysis of the CRD confirmed that, at physiological pH, the protein is predominantly a monomer (Fig. 6*B*). Previously reported structures of a fragment of BDCA-2 revealed a substantially different arrangement of the CRD, in which a dimer is formed by a domain swap between loops in the region that is found to contain the Ca^2+^-binding site in the structure reported here (14). Because those crystals were obtained in the absence of Ca^2+^, it was suggested that they represent an alternative, inactive form of the protein that might form under some conditions. The current results support the interpretation that the previous structures do not represent the ligand-binding conformation of BDCA-2.

##### Binding of BDCA-2 to Oligosaccharide Ligands

**FIGURE 7.**
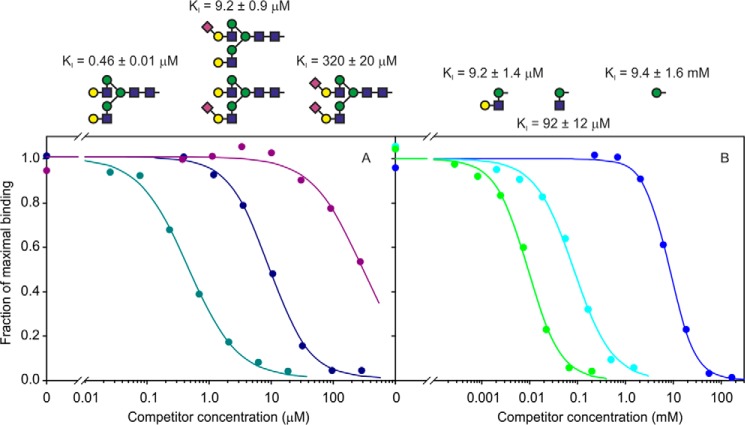
**Quantification of oligosaccharide binding to BDCA-2.** Binding competition assays were conducted with biotin-tagged CRD from BDCA-2 immobilized on streptavidin-coated plates, using ^125^I-Man-BSA as reporter ligand. *A*, competition with biantennary glycans released from egg yolk glycopeptide and treated with mild acid to remove sialic acid residues. *B*, competition with synthetic di- and trisaccharides. Glycan structures are encoded as in Fig. 3. The values of *K_I_* are presented as means ± standard deviations for two to four independent assays.

Quantitative information on the roles of additional sugars beyond mannose in the binding of complex *N*-linked glycans to BDCA-2 was obtained using the binding competition assay. Natural biantennary ligands were prepared by release of the oligosaccharide portion of the egg yolk glycopeptide with protein *N*-glycosidase and partial desialylation by mild acid treatment. Ion exchange chromatography was used to separate the starting disialylated oligosaccharide from monosialylated forms and the fully desialylated forms. Competition assays revealed a nearly 1000-fold increase in affinity upon removal of the two sialic acid residues (Fig. 7*A*), confirming that the CRD is selective for desialylated structures. The apparent affinity for the asialo biantennary glycan is nearly 20,000-fold higher than the affinity for mannose, reflecting the importance of additional sugars in the binding interaction.

The role of individual sugars was further dissected with synthetic di- and trisaccharides corresponding to parts of the Galβ1–4GlcNAcβ1–2Man epitope identified in the glycan array analysis. The addition of GlcNAc to mannose increases the affinity by 100-fold, whereas addition of galactose increases the affinity by a further 10-fold (Fig. 7*B*). These results are consistent with the finding on the glycan array, which showed binding of BDCA-2 to glycans bearing two terminal GlcNAcβ1–2Man structures, whereas those with a single GlcNAcβ1–2Man terminus did not bind significantly above background. On the other hand, the presence of a single terminal Galβ1–4GlcNAcβ1–2Man structure is sufficient for strong binding. These results indicate that interactions with both GlcNAc and galactose probably contribute to the strong affinity of the galactose-terminated complex *N*-linked glycans.

**FIGURE 8.**
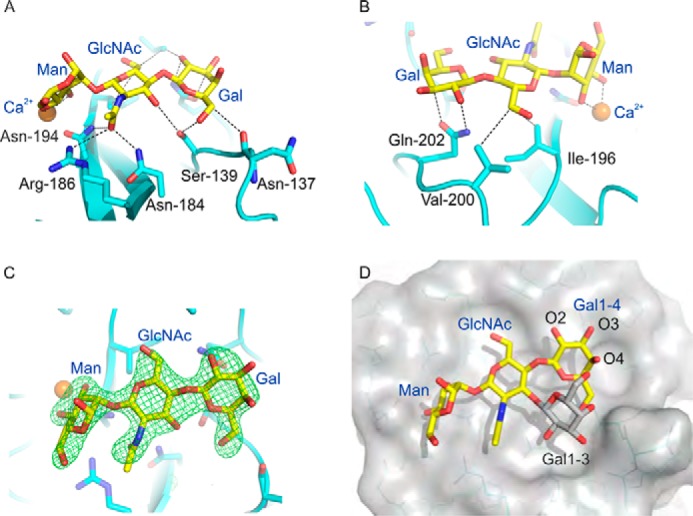
**Structural analysis of extended binding site in BDCA-2.** *A* and *B*, BDCA-2 complex with Galβ1–4GlcNAcβ1–2Man showing interactions between the protein and the GlcNAc and Gal moieties of the elongated carbohydrate. *C*, *F*_o_ − *F*_c_ map at 3.0 σ (*green*), showing Galβ1–4GlcNAcβ1–2Man at the binding site. *D*, surface representation of BDCA-2 with bound Galβ1–4GlcNAcβ1–2Man depicted as *sticks* with *yellow* carbon atoms. A galactose residue in β1–3 linkage to the GlcNAc, shown with *gray* carbon atoms, has been modeled to show that it can be accommodated in the binding site. Ca^2+^ is shown in *orange*, oxygen atoms are *red*, and nitrogen atoms are *blue*.

To investigate the arrangement of an oligosaccharide in the binding site of the CRD from BDCA-2, crystals were prepared in the presence of the trisaccharide ligand. The structure reveals that, as proposed, the mannose residue is located in the primary binding site, in the more common orientation seen in the crystals with α-methyl mannoside (Fig. 8). The other two sugar residues occupy a shallow groove, the width of which accommodates the 1–4-linked sugar but that could potentially hold an even larger oligosaccharide adjacent to the primary binding site. The temperature factors for the ligand are not significantly different from those for the protein (Table 2), indicating that the oligosaccharide is fully occupied in the structure, and the electron density (Fig. 8*C*) unambiguously defines the bound oligosaccharide orientation and conformation. On one side of the GlcNAc, the carbonyl group in the acetamido substituent at position 2 is engaged in hydrogen bonds with the side chains of Asn-184, Arg-186, and Asn-194. This last side chain is also a Ca^2+^ ligand and part of the primary binding site (Fig. 8*A*). On the other side of the GlcNAc, residues Val-200 and Ile-196 make van der Waals contacts with the exocyclic 6-hydroxymethyl group (Fig. 8*B*). For the galactose, the side chain of Ser-139 is hydrogen-bonded to OH-6, and the side chain of Gln-202 forms hydrogen bonds with OH-2 and makes van der Waals contact with C3 (Fig. 8, *A* and *B*).

**FIGURE 9.**
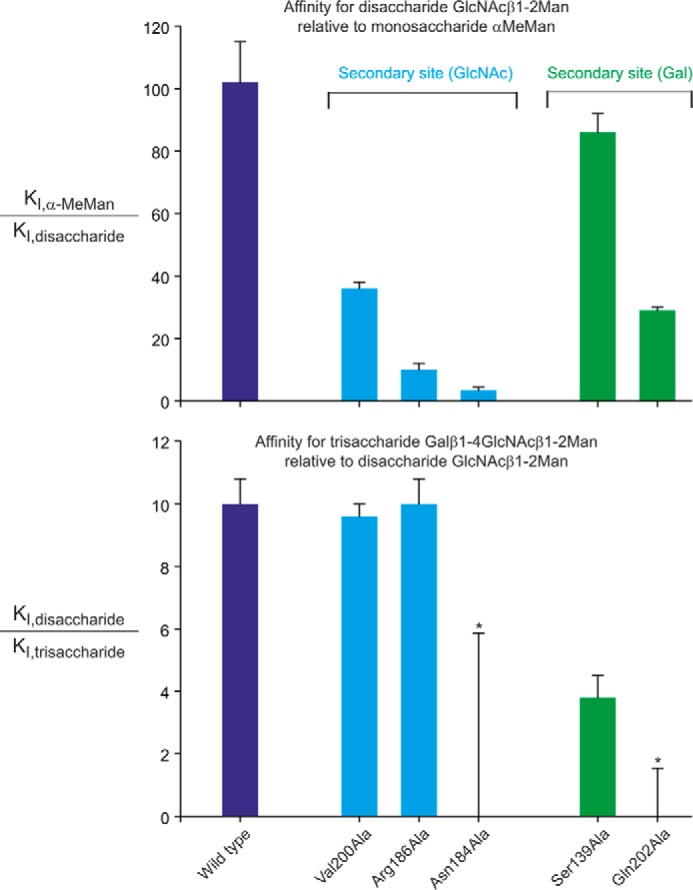
**Mutational analysis of extended binding site in BDCA-2.** Affinities for disaccharide and trisaccharide ligands are compared for the wild type and mutant CRDs, by plotting ratios of inhibition constants for oligosaccharides and α-methyl mannoside in the solid phase binding assay. The values of means ± standard deviations for two to four assays are shown. For the two measurements marked with an *asterisk*, no inhibition was seen at the highest trisaccharide concentration that could be tested (200 μm), so the *error bars* represent the highest possible ratio of affinities, based on an estimated *K_I_* value > 500 μm.

The importance of the interactions observed in the crystal structure was confirmed by mutating residues that make contacts with the GlcNAc and galactose residues in the extended binding site. In the GlcNAc site, changing Val-200 or Arg-186 to alanine results in reductions of 3- and 10-fold in the affinities for both di- and trisaccharide ligands GlcNAcβ1–2Man and Galβ1–4GlcNAcβ1–2Man, whereas the mutation N184A almost completely abolishes enhanced binding to both ligands (Fig. 9). The first two of these mutations preserve the preferential binding of the trisaccharide ligand compared with the disaccharide ligand, which is consistent with the fact that the galactose-binding portion of the site has not been changed. Because of the very large reduction in overall binding affinity for the N184A mutation, it was not possible to test the trisaccharide ligand at sufficiently high concentration to confirm whether it still binds better than the disaccharide.

Mutation of residues that interact with the galactose would be expected to affect binding of the Galβ1–4GlcNAcβ1–2Man trisaccharide more than the disaccharide lacking galactose. In line with this prediction, mutation of either Ser-139 or Gln-202 to alanine results in a significant loss of relative affinity for the trisaccharide compared with the disaccharide (Fig. 9). Although Ser-139 in monomer A is close enough to the 3-OH group of GlcNAc to make a hydrogen bond, in monomer B, it interacts only with the galactose residue. The fact that the S139A mutation has little effect on binding of the disccharide ligand suggests that the potential hydrogen bond to GlcNAc does not contribute significantly to binding of the disaccharide. The finding that the Q202A mutation results in a decrease in affinity for the disaccharide compared with mannose suggests that when the disaccharide lacking galactose is bound, GlcNAc may shift slightly to interact with Gln-202 rather than Ser-139. In any case, the characteristics of the mutant CRDs support the conclusion that the arrangement of the trisaccharide observed in the crystal structure accurately reflects the mode of interaction between the CRD and the ligand in solution.

The arrangement of the binding site is consistent with the range of ligands on the glycan array that bind BDCA-2. For example, the broad canyon adjacent to the primary binding site would accommodate galactose linked 1–3 to the GlcNAc residue, as well as the 1–4-linked galactose observed in the crystal structure (Fig. 8*D*). The 4-OH group of the 1–4-linked galactose projects away from the surface of the protein, which explains why addition of a further galactose at this position is tolerated. In contrast, addition of sugars to the 2-, 3-, or 6-OH groups would result in steric clashes. For example, overlaying the NeuAcα2–3Galβ1–4GlcNAc and NeuAcα2–6Galβ1-4GlcNAc portions of various glycans observed in crystal structures of glycoproteins indicates that all of the observed conformations of the sialic acid residues would clash with the protein surface. Similarly, the 3-OH group of GlcNAc points toward the protein surface, so addition of fucose at this position would also result in a steric clash.

Ligands with the Galβ1–4GlcNAcβ1–2Man epitope on the 1–3 arm of bi-, tri-, and tetra-antennary glycans bind better than those in which the epitope is present on the 1–6 arm. Modeling with the glycan from IgG (Protein Data Bank entry 1HZH) and other biantennary glycans reveals that in all cases, the Galβ1–4GlcNAcβ1–2Man epitope on the 1–3 arm can be accommodated in the binding site of BDCA-2, with both the 1–6 arm and the chitobiose core projecting away from the protein. In contrast, for most of the available structures, there is a clash between the protein surface and the chitobiose core of the oligosaccharide when the trisaccharide binding epitope on the 1–6 arm is positioned in the binding site, although in one case in which the 1–6 arm assumes an alternative conformation (Protein Data Bank entry 1SLA), the terminal trisaccharide on this arm can bind. Thus, the less favorable interaction with the 1–6 branch probably results from the need for the oligosaccharide to assume a less favored orientation in order for this binding to occur. Particularly weak binding is observed for glycans that are branched on the 1–3 arm, including tetra-antennary glycans, because these structures contain linkages to the 4 position of the mannose on this branch, which blocks binding to the favored terminal structure.

## Discussion

The results of the analysis reported here confirm and extend earlier reports that BDCA-2 binds to galactose-terminated biantennary glycans, by defining an epitope found on a limited number of bi- and triantennary glycans that are not fully sialylated. The results also reconcile an apparent discrepancy between the nature of these ligands and the predicted specificity of the primary binding site for mannose-type ligands, because the specificity for galactose-terminated glycans comes from secondary site interactions rather than binding to the Ca^2+^ in the primary binding site.

These findings help to put previously described structures of BDCA-2 in perspective. In these structures, formed under low Ca^2+^ conditions, pairs of CRDs undergo domain swapping (14). As was noted, these structures are unlikely to be the ligand-binding conformation at the cell surface because of the absence of bound Ca^2+^, but they may represent forms that would occur under some conditions. A similar domain-swapped structure has been observed for another sugar-binding C-type CRD, domain 4 of the mannose receptor (34), and a domain-swapped dimer with somewhat different geometry has been found for snake venom IX/X-binding protein, which contains a C-type lectin-like domain that does not bind sugars (35). The finding that only a single Ca^2+^ binds to the CRD from BDCA-2 may mean that it is more susceptible to such a reorganization.

The arrangement of galactose-terminated glycans in the binding site of BDCA-2 contrasts with the arrangement of the asialoglycoprotein receptor, which can bind some of the same glycans, but in which the primary binding is through the galactose residue (36, 37). A similar situation is observed for binding of Lewis^x^ to DC-SIGN and the scavenger receptor C-type lectin, with the former binding through fucose in the primary binding site and galactose in an adjacent secondary site, whereas the latter binds with galactose in the primary binding site and fucose making secondary interactions (5, 38).

**FIGURE 10.**
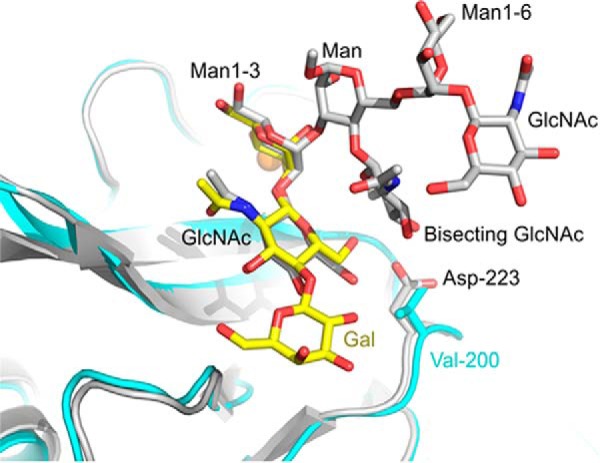
**Comparison of ligand binding sites in human BDCA-2 and mouse DCIR2.** The structure of mouse DCIR2 is taken from Protein Data Bank entry 3VYK and is shown in *gray*. BDCA-2 is shown in *cyan*. In the superposed structures, carbon atoms of Galβ1–4GlcNAcβ1–2Man bound to BDCA-2 are in *yellow*, and carbon atoms of the biantennary glycan with bisecting GlcNAc, bound to DCIR2, are in *gray*. Ca^2+^ is shown in *orange*, oxygen atoms in *red*, and nitrogen atoms are *dark blue*.

It is interesting to compare the structure of human BDCA-2 with the structure of the mouse DCIR2, which binds to biantennary *N*-linked glycans with bisecting GlcNAc residues attached to the 4-OH group of the core mannose residue (39). Superficially, these specificities seem very different, but the binding epitope for DCIR2 includes the GlcNAcβ1–2Man that binds to BDCA-2, and a similar arrangement of these two residues is seen in the two proteins (Fig. 10). Similarities are seen both in the position of mannose in the primary binding site and the contacts made with GlcNAc in the secondary binding site. In addition to these interactions, specificity for the bisecting GlcNAc is achieved in DCIR2 by interaction of this residue with Glu-202 and Asp-223, which correspond to Glu-178 and Val-200 in BDCA-2. The absence of the aspartic acid residue in BDCA-2 would lead to destabilization of this binding interaction.

Defining the types of ligands that bind to BDCA-2 provides some insights into potential biological functions of the receptor. For example, the structure explains why BDCA-2 does not bind to glycans bearing terminal sialic acid residues, because addition of NeuAc at either the 3- or 6-OH group of galactose results in steric clashes. This arrangement contrasts with the asialoglycoprotein receptor, in which the 3-OH of galactose is buried in the primary binding site so that ligands with α2–3 linked sialic acid are excluded, whereas the 6-OH group is exposed, so that sialylation is tolerated on this position (36, 37).

The preference for desialylated glycans might seem at odds with the rapid clearance of glycoproteins bearing such glycans by the hepatic asialoglycoprotein receptor. However, there are notable examples of proteins, including serum IgG, that largely lack terminal sialic acid. As demonstrated here, this unusual glycosylation allows binding of IgG to BDCA-2 with an affinity of ∼1 μm. Because the glycans on IgG are located on the constant Fc domain, BDCA-2 could function as a previously unrecognized Fc receptor with an affinity similar to that observed for FcγRII and FcγRIII (40). Cross-linking of BDCA-2 has been demonstrated to block signaling initiated in response to Toll-like receptors in plasmacytoid dendritic cells, resulting in decreased type 1 interferon secretion (6). Interferon production is important in stimulating the normal immune response, and high levels can lead to autoimmunity (41). Thus, the finding that serum IgG is a ligand for BDCA-2 suggests a potential regulatory mechanism in response to rising levels of serum IgG, which would tend to dampen down the response once IgG has reached sufficient concentration in the blood. Because BDCA-2 is expressed in humans but not in mice, the consequences of BDCA-2 binding to IgG or other serum glycoproteins cannot be assessed with mouse models, and its properties may account for some differences in the behavior of the human and mouse immune systems.

## Supplementary Material

Supplemental Data

## Footnotes

3 The abbreviations used are:

BDCA-2: blood dendritic cell antigen 2
CRD: carbohydrate recognition domain
DCIR: dendritic cell immunoreceptor.
