# Supplementary material for: A Novel Mechanism for Binding of Galactose-terminated Glycans by the C-type Carbohydrate Recognition Domain in Blood Dendritic Cell Antigen 2
Source: J Biol Chem. 2015 May 20;290(27):16759–71. doi: 10.1074/jbc.M115.660613 (PMC4505424; doi:10.1074/jbc.M115.660613)
Supplement: Supplemental Data [file supp_290_27_16759__index.html]

A novel mechanism for binding of galactose-terminated glycans by the C-type carbohydrate-recognition domain in blood dendritic cell antigen 2 — A Novel Mechanism for Binding of Galactose-terminated Glycans by the C-type Carbohydrate Recognition Domain in Blood Dendritic Cell Antigen 2 — BDCA-2 Glycan-binding Site — Supplemental Data 

# A Novel Mechanism for Binding of Galactose-terminated Glycans by the C-type Carbohydrate Recognition Domain in Blood Dendritic Cell Antigen 2

## Supplemental Data

- Supplemental Information (.pdf, 179 KB) - Supplemental Table S1 and Figures S1 and S2
