# Supplementary material for: A Novel Mechanism for Binding of Galactose-terminated Glycans by the C-type Carbohydrate Recognition Domain in Blood Dendritic Cell Antigen 2
Source: J Biol Chem. 2015 May 20;290(27):16759–71. doi: 10.1074/jbc.M115.660613 (PMC4505424; doi:10.1074/jbc.M115.660613)
Supplement: Supplemental Data [file supp_M115.660613_jbc.M115.660613-1.pdf]

Supplemental Information

Blood Dendritic Cell Antigen 2 Binds Selectively to Galactose-terminated Glycans through a Mannose-type Binding Site

Hadar Feinberg, Sabine A. F. Jégouzo, Tabassum Dungarwalla,
Kurt Drickamer, William I. Weis, and Maureen E. Taylor

Table S1. Screening of BDCA-2 on CFG Glycan Array version 5.1.

Figure S1. Mass spectrometry of BDCA-2 trisaccharide ligand.

Figure S2. Proton NMR spectrum of BDCA-2 trisaccharide ligand.

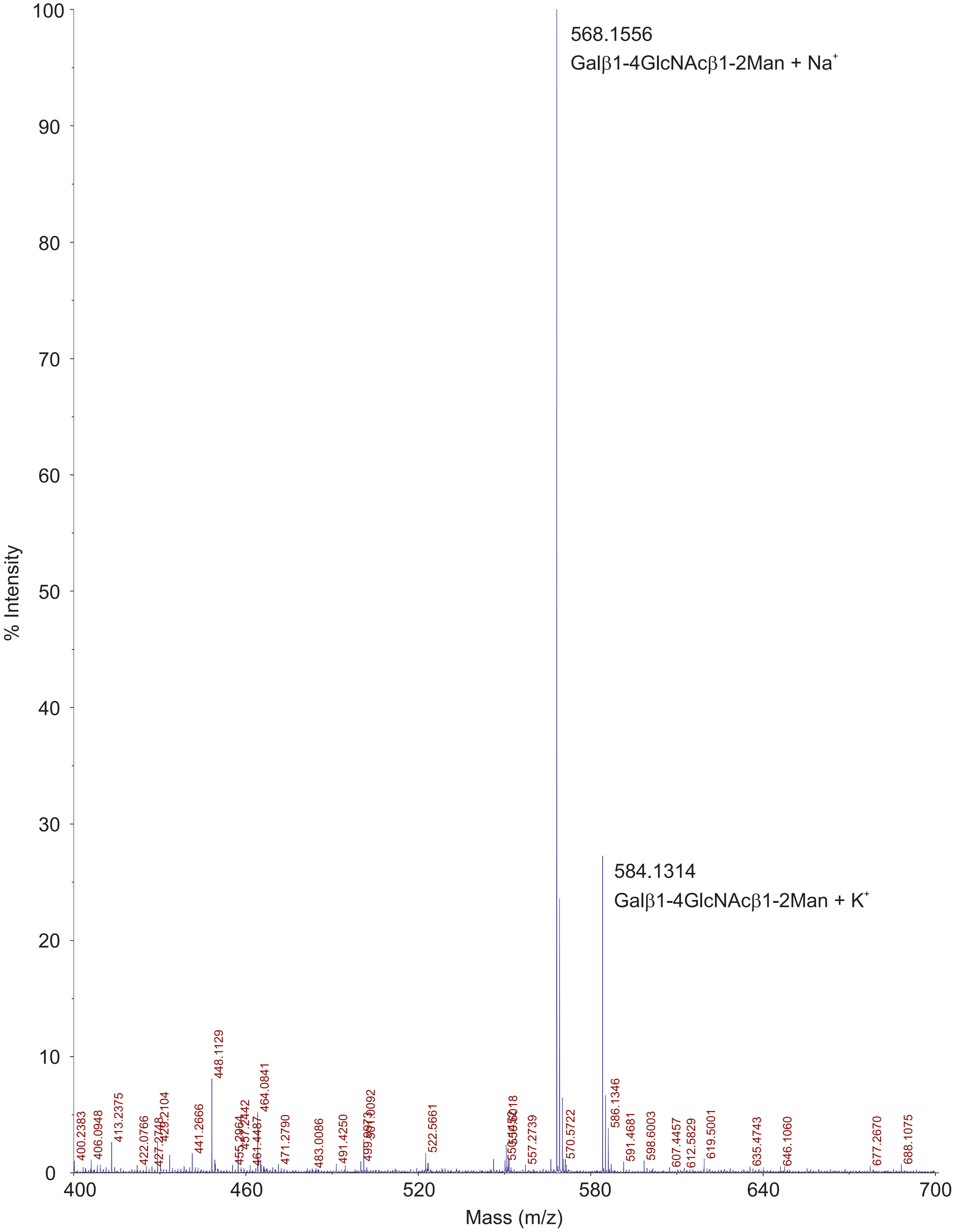

Figure S1. Mass spectrometry of synthetic trisaccharide. Samples were prepared by spotting together equal volumes of matrix, 2,5-dihydroxybenzoic acid as a 10 mg/ml solution 80% methanol, and sugar derivative, dissolved at approximately 1 mg/ml in water, on a target plate. Mass spectrometry was performed on an Applied Biosystems 4800 matrix assisted-laser desorption time-of-flight mass spectrometer.

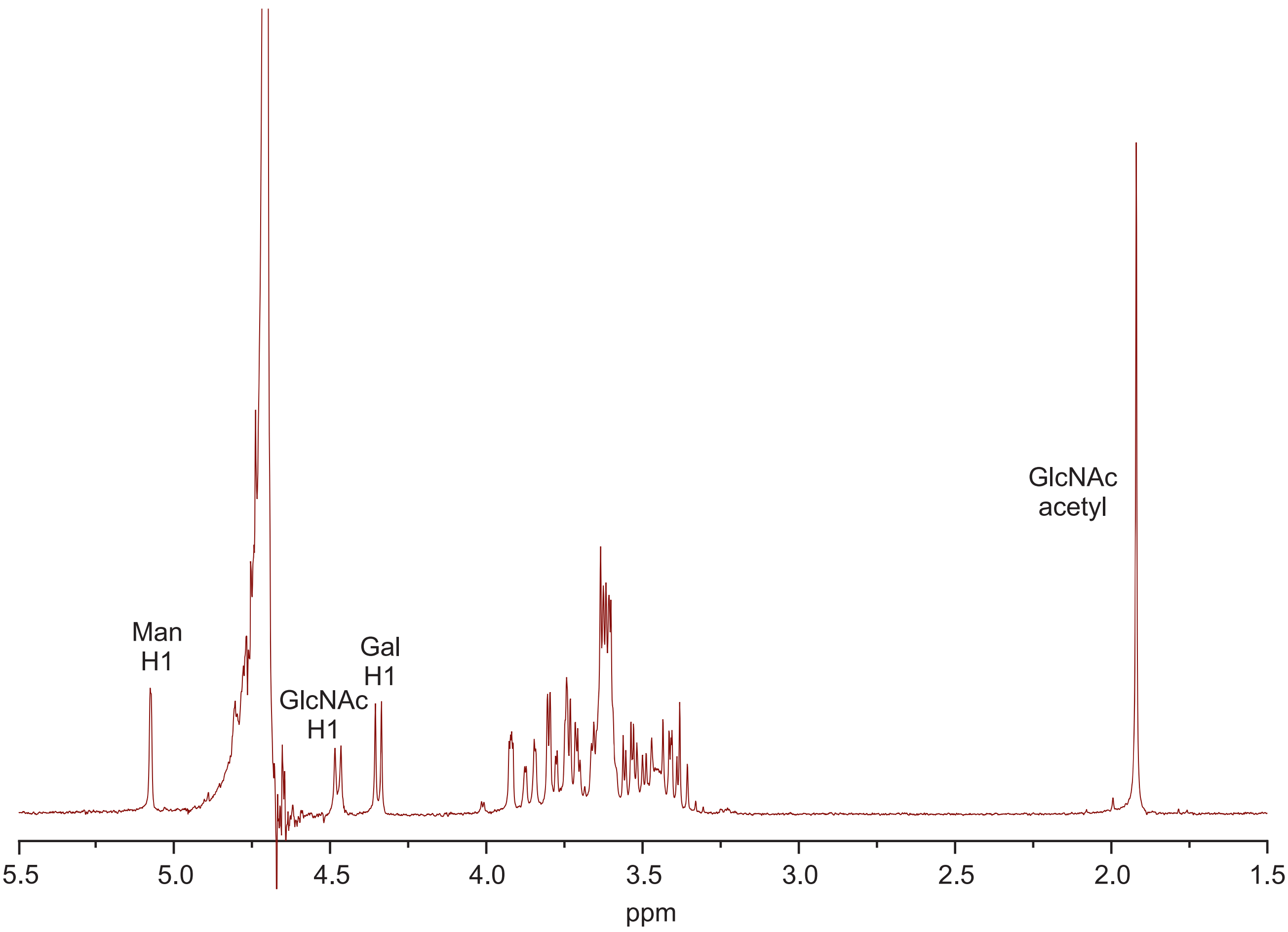

Figure S2. Proton NMR spectra of trisaccharide ligand for BDCA-2. Sample for NMR spectra, dissolved in at approximately 2 mg/ml, was analyzed on a Bruker 400 MHz spectrometer.

Table S1. Screening of BDCA-2 on CFG Glycan Array version 5.1

Glycan number	Glycan structure	Average RFU	Standard Deviation
301	Neu5Acα2-6Galβ1-4GlcNAcβ1-2Manα1-6(Galβ1-4GlcNAcβ1-2Manα1-3)Manβ1-4GlcNAcβ1-4GlcNAcβ-Sp12	5886	161
485	Galβ1-4GlcNAcβ1-6(Galβ1-4GlcNAcβ1-2)Manα1-6(Galβ1-4GlcNAcβ1-2Manα1-3)Manβ1-4GlcNAcβ1-4(Fucα1-6)GlcNAcβ-Sp24	4117	162
404	Galα1-4Galβ1-4GlcNAcβ1-2Manα1-6(Galα1-4Galβ1-4GlcNAcβ1-2Manα1-3)Manβ1-4GlcNAcβ1-4GlcNAcβ-Sp24	4069	138
324	Galβ1-3GlcNAcβ1-2Manα1-6(Galβ1-3GlcNAcβ1-2Manα1-3)Manβ1-4GlcNAcβ1-4GlcNAcβ-Sp19	3985	328
54	Galβ1-4GlcNAcβ1-2Manα1-6(Galβ1-4GlcNAcβ1-2Manα1-3)Manβ1-4GlcNAcβ1-4GlcNAcβ-Sp12	3667	140
458	Galβ1-4GlcNAcβ1-6(Galβ1-4GlcNAcβ1-2)Manα1-6(Galβ1-4GlcNAcβ1-2Manα1-3)Manβ1-4GlcNAcβ1-4GlcNAcβ-Sp19	3502	296
397	GlcNAcβ1-2Manα1-6(Galβ1-4GlcNAcβ1-2Manα1-3)Manβ1-4GlcNAcβ1-4GlcNAc-Sp12	3461	245
349	Galβ1-4GlcNAcβ1-2Manα1-3Manβ1-4GlcNAcβ1-4GlcNAc-Sp12	2918	324
531	Galβ1-4GlcNAcβ1-2 Manα1-6(GlcNAcβ1-4)(Galβ1-4GlcNAcβ1-2Manα1-3)Manβ1-4GlcNAcβ1-4(Fucα1-6)GlcNAc-Sp21	2036	97
353	Galβ1-4GlcNAcβ1-2Manα1-6(Galβ1-4GlcNAcβ1-2Manα1-3)Manβ1-4GlcNAcβ1-4(Fucα1-6)GlcNAcβ-Sp22	1880	285
351	Manα1-6(Galβ1-4GlcNAcβ1-2Manα1-3)Manβ1-4GlcNAcβ1-4GlcNAcβ-Sp12	1828	87
398	Galβ1-4GlcNAcβ1-2Manα1-6(GlcNAcβ1-2Manα1-3)Manβ1-4GlcNAcβ1-4GlcNAc-Sp12	1726	86
428	Galβ1-3GlcNAcβ1-6(Galβ1-3GlcNAcβ1-2)Manα1-6(Galβ1-3GlcNAcβ1-2Manα1-3)Manβ1-4GlcNAcβ1-4GlcNAcβ-Sp19	947	68
53	GlcNAcβ1-2Manα1-6(GlcNAcβ1-2Manα1-3)Manβ1-4GlcNAcβ1-4GlcNAcβ-Sp13	838	137
452	Neu5Acα2-8Neu5Acα2-3Galβ1-3GalNAcβ1-4(Neu5Acα2-8Neu5Acα2-3)Galβ1-4Glcβ-Sp0	830	71
354	Galβ1-3GlcNAcβ1-2Manα1-6(Galβ1-3GlcNAcβ1-2Manα1-3)Manβ1-4GlcNAcβ1-4(Fucα1-6)GlcNAcβ-Sp22	712	294
319	Galβ1-4GlcNAcβ1-2Manα1-6(Neu5Acα2-6Galβ1-4GlcNAcβ1-2Manα1-3)Manβ1-4GlcNAcβ1-4GlcNAcβ-Sp12	631	23
350	Galβ1-4GlcNAcβ1-2Manα1-6Manβ1-4GlcNAcβ1-4GlcNAc-Sp12	544	108
364	Galβ1-4GlcNAcβ1-2Manα1-6(Manα1-3)Manβ1-4GlcNAcβ1-4GlcNAcβ-Sp12	506	103
476	Galβ1-3GlcNAcβ1-2Manα1-6(GlcNAcβ1-4)(Galβ1-3GlcNAcβ1-2Manα1-3)Manβ1-4GlcNAcβ1-4GlcNAcβ-Sp21	373	54
435	Galβ1-4GlcNAcβ1-2Manα1-6(GlcNAcβ1-4)(Galβ1-4GlcNAcβ1-2Manα1-3)Manβ1-4GlcNAcβ1-4GlcNAc-Sp21	366	104
437	Galβ1-4GlcNAcβ1-6(Galβ1-4GlcNAcβ1-2)Manα1-6(GlcNAcβ1-4)(Galβ1-4GlcNAcβ1-2Manα1-3)Manβ1-4GlcNAcβ1-4GlcNAc-Sp21	294	30
421	GlcNAcβ1-2(GlcNAcβ1-6)Manα1-6(GlcNAcβ1-2Manα1-3)Manβ1-4GlcNAcβ1-4GlcNAcβ-Sp19	262	13
52	GlcNAcβ1-2Manα1-6(GlcNAcβ1-2Manα1-3)Manβ1-4GlcNAcβ1-4GlcNAcβ-Sp12	255	88

369	Galβ1-4GlcNAcβ1-2Manα1-6(Galβ1-4GlcNAcβ1-4(Galβ1-4GlcNAcβ1-2)Manα1-3)Manβ1-4GlcNAcβ1-4GlcNAc-Sp21	254	10
387	Galβ1-4GlcNAcβ1-6(Galβ1-4GlcNAcβ1-2)Manα1-6(Galβ1-4GlcNAcβ1-4(Galβ1-4GlcNAcβ1-2)Manα1-3)Manβ1-4GlcNAcβ1-4GlcNAcβ-Sp21	219	22
524	Galβ1-3GlcNAcβ1-2Manα-Sp0	171	30
532	Galβ1-4GlcNAcβ1-2 Manα1-6(Galβ1-4GlcNAcβ1-4)(Galβ1-4GlcNAcβ1-2Manα1-3)Manβ1-4GlcNAcβ1-4(Fucα1-6)GlcNAc-Sp21	171	125
541	Galβ1-4GlcNAcβ1-3Galβ1-4GlcNAcβ1-2Manα1-6(Galβ1-4GlcNAcβ1-3Galβ1-4GlcNAcβ1-2Manα1-3)Manβ1-4GlcNAcβ1-4GlcNAcβ-Sp12	109	8
215	Manα1-6(Manα1-3)Manα1-6(Manα1-2Manα1-3)Manβ1-4GlcNAcβ1-4GlcNAcβ-Sp12	106	19
474	Neu5Acα2-3Galβ1-3GlcNAcβ1-6(Neu5Acα2-3Galβ1-3GlcNAcβ1-2)Manα1-6(Neu5Acα2-3Galβ1-3GlcNAcβ1-2Manα1-3)Manβ1-4GlcNAcβ1-4GlcNAcβ-Sp19	105	22
526	Neu5Acα2-3Galβ1-3GlcNAcβ1-2Manα-Sp0	100	8
501	Fucα1-2(6S)Galβ1-3(6S)GlcNAcβ-Sp0	100	49
204	GlcAβ1-6Galβ-Sp8	96	17
478	Neu5Acα2-3Galβ1-4GlcNAcβ1-2Manα-Sp0	92	18
547	Galβ1-4GlcNAcβ1-3Galβ1-4GlcNAcβ1-3Galβ1-4GlcNAcβ1-2Manα1-6(Galβ1-4GlcNAcβ1-3Galβ1-4GlcNAcβ1-3Galβ1-4GlcNAcβ1-2Manα1-3)Manβ1-4GlcNAcβ1-4GlcNAcβ-Sp12	90	86
308	Neu5Acα2-6Galβ1-4GlcNAcβ1-2Manα1-6(GlcNAcβ1-2Manα1-3)Manβ1-4GlcNAcβ1-4GlcNAcβ-Sp12	90	20
358	KDNα2-3Galβ1-4Glc-Sp0	88	30
365	Fucα1-4(Galβ1-3)GlcNAcβ1-2Manα1-6(Fucα1-4(Galβ1-3)GlcNAcβ1-2Manα1-3)Manβ1-4GlcNAcβ1-4(Fucα1-6)GlcNAcβ-Sp22	88	36
418	GalNAcα1-3(Fucα1-2)Galβ1-4(Fucα1-3)GlcNAcβ1-3GalNAc-Sp14	79	28
217	Manβ1-4GlcNAcβ-Sp0	79	24
56	Neu5Acα2-6Galβ1-4GlcNAcβ1-2Manα1-6(Neu5Acα2-6Galβ1-4GlcNAcβ1-2Man-α1-3)Manβ1-4GlcNAcβ1-4GlcNAcβ-Sp21	75	4
249	Neu5Acα2-3Galβ1-3GlcNAcβ-Sp0	65	22
508	Galβ1-4GlcNAcβ1-6(Galβ1-4GlcNAcβ1-2)Manα1-6(GlcNAcβ1-4)Galβ1-4GlcNAcβ1-4(Gal β1-4GlcNAcβ1-2)Manα1-3)Manβ1-4GlcNAcβ1-4(Fucα1-6)GlcNAc-Sp21	61	28
375	Fucα1-4(Fucα1-2Galβ1-3)GlcNAcβ1-2Manα1-3(Fucα1-4(Fucα1-2Galβ1-3)GlcNAcβ1-2Manα1-3)Manβ1-4GlcNAcβ1-4GlcNAcβ-Sp19	60	14
372	Galα1-3Galβ1-4(Fucα1-3)GlcNAcβ1-2Manα1-6(Galα1-3Galβ1-4(Fucα1-3)GlcNAcβ1-2Manα1-3)Manβ1-4GlcNAcβ1-4GlcNAcβ-Sp20	60	28
395	Galα1-3Galβ1-3(Fucα1-4)GlcNAcβ1-2Manα1-6(Galα1-3Galβ1-3(Fucα1-4)GlcNAcβ1-2Manα1-3)Manβ1-4GlcNAcβ1-4GlcNAc-Sp19	59	25

475	GlcNAcβ1-6(GlcNAcβ1-2)Manα1-6(GlcNAcβ1-2Manα1-3)Manβ1-4GlcNAcβ1-4(Fucα1-6)GlcNAcβ-Sp24	59	46
352	GlcNAcβ1-2Manα1-6(GlcNAcβ1-2Manα1-3)Manβ1-4GlcNAcβ1-4(Fucα1-6)GlcNAcβ-Sp22	57	8
416	Fucα1-2Galβ1-4(Fucα1-3)GlcNAcβ1-3GalNAcα-Sp14	56	17
533	Fucα1-4(Galβ1-3)GlcNAcβ1-2 Manα-Sp0	56	5
449	Fucα1-2Galβ1-4GlcNAcβ1-6(Fucα1-2Galβ1-4GlcNAcβ1-3)GalNAc-Sp14	55	14
453	GalNAcβ1-4Galβ1-4Glcβ-Sp0	55	14
427	Galα1-3(Fucα1-2)Galβ1-4GlcNAcβ1-2Manα1-6(Galα1-3(Fucα1-2)Galβ1-4GlcNAcβ1-2Manα1-3)Manβ1-4GlcNAcβ1-4(Fucα1-6)GlcNAcβ-Sp22	55	27
469	Glcα1-6Glcα1-6Glcα1-6Glcβ-Sp10	54	7
211	Manα1-2Manα1-6(Manα1-3)Manα1-6(Manα1-2Manα1-2Manα1-3)Manβ1-4GlcNAcβ1-4GlcNAcβ-Sp12	54	5
480	Neu5Acα2-6Galβ1-4GlcNAcβ1-6GalNAcα-Sp14	53	22
361	Fucα1-2Galβ1-4GlcNAcβ1-2Manα1-6(Fucα1-2Galβ1-4GlcNAcβ1-2Manα1-3)Manβ1-4GlcNAcβ1-4GlcNAcβ-Sp20	52	13
342	GlcNAcα1-4Galβ1-4GlcNAcβ1-3Galβ1-4(Fucα1-3)GlcNAcβ1-3Galβ1-4(Fucα1-3)GlcNAcβ-Sp0	51	14
518	(6P)Galβ1-4GlcNAcβ-SP0	51	33
522	Galα1-3(Fucα1-2)Galβ1-4GlcNAcβ1-2Manα-Sp0	49	19
360	Fucα1-2Galβ1-3GlcNAcβ1-2Manα1-6(Fucα1-2Galβ1-3GlcNAcβ1-2Manα1-3)Manβ1-4GlcNAcβ1-4GlcNAcβ-Sp20	49	6
412	Neu5Acα2-3Galβ1-3GalNAcβ1-4(Neu5Acα2-8Neu5Acα2-3)Galβ1-4Glcβ-Sp0	49	15
527	Galα1-3Galβ1-3GlcNAcβ1-2Manα-Sp0	48	28
530	GlcNAcβ1-2 Manα1-6(GlcNAcβ1-4)(GlcNAcβ1-2Manα1-3)Manβ1-4GlcNAcβ1-4(Fucα1-6)GlcNAc-Sp21	48	19
55	Neu5Acα2-6Galβ1-4GlcNAcβ1-2Manα1-6(Neu5Acα2-6Galβ1-4GlcNAcβ1-2Manα1-3)Manβ1-4GlcNAcβ1-4GlcNAcβ-Sp12	48	42
545	GlcNAcβ1-3Galβ1-4GlcNAcβ1-3Galβ1-4GlcNAcβ1-2Manα1-6(GlcNAcβ1-3Galβ1-4GlcNAcβ1-3Galβ1-4GlcNAcβ1-2Manα1-3)Manβ1-4GlcNAcβ1-4GlcNAcβ-Sp12	48	17
426	Fucα1-2Galβ1-3GlcNAcβ1-2Manα1-6(Fucα1-2Galβ1-3GlcNAcβ1-2Manα1-3)Manβ1-4GlcNAcβ1-4(Fucα1-6)GlcNAcβ-Sp22	48	11
413	Galα1-3(Fucα1-2)Galβ1-4GlcNAcβ1-3GalNAcα-Sp14	46	25
450	Galα1-3(Fucα1-2)Galβ1-4GlcNAcβ1-6(Galα1-3(Fucα1-2)Galβ1-4GlcNAcβ1-3)GalNAc-Sp14	46	30
423	Galα1-3(Fucα1-2)Galβ1-3GlcNAcβ1-3GalNAc-Sp14	44	28
274	Neu5Acα2-6Galβ-Sp8	44	19
467	Galα1-3(Fucα1-2)Galβ1-3GalNAcα-Sp8	43	6
313	Manα1-6Manβ-Sp10	43	5
362	Fucα1-2Galβ1-4(Fucα1-3)GlcNAcβ1-2Manα1-6(Fucα1-2Galβ1-4(Fucα1-3)GlcNAcβ1-2Manα1-3)Manβ1-4GlcNAcβ1-4GlcNAβ-Sp20	42	21
229	Neu5Acα2-8Neu5Acα2-8Neu5Acα-Sp8	41	6
468	Galα1-3(Fucα1-2)Galβ1-3GalNAcβ-Sp8	41	13

51	Manα1-6(Manα1-3)Manβ1-4GlcNAcβ1-4GlcNAcβ-Sp13	40	21
537	Galα1-3(Fucα1-2)Galβ1-3GalNAcβ1-3Galα1-4Galβ1-4Glc-Sp21	40	24
560	Galα1-3Galβ1-4GlcNAcβ1-2Manα1-6(Galα1-3Galβ1-4GlcNAcβ1-2Manα1-3)Manβ1-4GlcNAcβ1-4GlcNAc-Sp24	39	8
436	Galβ1-4GlcNAcβ1-2Manα1-6(GlcNAcβ1-4)(Galβ1-4GlcNAcβ1-4(Galβ1-4GlcNAcβ1-2)Manα1-3)Manβ1-4GlcNAcβ1-4GlcNAc-Sp21	39	15
572	Galβ1-3GlcNAcβ1-3Galβ1-4GlcNAcβ1-6(Galβ1-3GlcNAcβ1-3Galβ1-4GlcNAβ1-2)Manα1-6(Galβ1-3GlcNAcβ1-3Galβ1-4GlcNAcβ1-2Manα1-3)Manβ1-4GlcNAcβ1-4(Fucα1-6)GlcNAcβ-Sp24	39	22
605	Neu5Acα2-6Galβ1-4GlcNAcβ1-3Galβ1-4GlcNAcβ1-6(Neu5Acα2-6Galβ1-4GlcNAcβ1-3Galβ1-4GlcNAcβ1-3)GalNAcα-Sp14	39	18
402	GalNAcα1-3GalNAcβ1-3Galα1-4Galβ1-4GlcNAcβ-Sp0	38	18
371	Galα1-3(Fucα1-2)Galβ1-4GlcNAcβ1-2Manα1-6(Galα1-3(Fucα1-2)Galβ1-4GlcNAcβ1-2Manα1-3)Manβ1-4GlcNAcβ1-4GlcNAcβ-Sp20	38	9
464	Neu5Acα2-6Galβ1-4GlcNAcβ1-4Manα1-6(GlcNAcβ1-4)(Neu5Acα2-6Galβ1-4GlcNAcβ1-4(Neu5Acα2-6Galβ1-4GlcNAcβ1-2)Manα1-3)Manβ1-4GlcNAcβ1-4GlcNAcβ-Sp21	37	12
346	Manα1-6(Neu5Acα2-6Galβ1-4GlcNAcβ1-2Manα1-3)Manβ1-4GlcNAcβ1-4GlcNAc-Sp12	37	18
234	Neu5Acα2-3Galβ1-3GalNAcβ1-4(Neu5Acα2-3)Galβ1-4Glcβ-Sp0	37	11
298	(6P)Glcβ-Sp10	37	20
571	Galβ1-3GlcNAcβ1-3Galβ1-4GlcNAcβ1-3Galβ1-4GlcNAcβ1-6(Galβ1-3GlcNAcβ1-3Galβ1-4GlcNAcβ1-3Galβ1-4GlcNAβ1-2)Manα1-6(Galβ1-3GlcNAcβ1-3Galβ1-4GlcNAcβ1-3Galβ1-4GlcNAcβ1-2Manα1-3)Manβ1-4GlcNAcβ1-4(Fucα1-6)GlcNAcβ-Sp24	36	12
394	Galα1-3Galβ1-3GlcNAcβ1-2Manα1-6(Galα1-3Galβ1-3GlcNAcβ1-2Manα1-3)Manβ1-4GlcNAcβ1-4GlcNAc-Sp19	36	11
363	Galα1-3Galβ1-4GlcNAcβ1-2Manα1-6(Galα1-3Galβ1-4GlcNAcβ1-2Manα1-3)Manβ1-4GlcNAcβ1-4GlcNAcβ-Sp20	36	4
379	GalNAcβ1-4GlcNAcβ1-2Manα1-6(GalNAcβ1-4GlcNAcβ1-2Manα1-3)Manβ1-4GlcNAcβ1-4GlcNAc-Sp12	36	13
451	GalNAcα1-3(Fucα1-2)Galβ1-4GlcNAcβ1-6(GalNAcα1-3(Fucα1-2)Galβ1-4GlcNAcβ1-3)GalNAc-Sp14	36	26
401	Galβ1-4(Fucα1-3)GlcNAcβ1-3GalNAcα-Sp14	35	16
41	(6P)Manα-Sp8	35	25
440	Galβ1-6Galβ-Sp10	35	39
587	GlcNAcβ1-3Galβ1-4GlcNAcβ1-3Galβ1-4GlcNAcβ1-3Galβ1-4GlcNAcβ1-3Galβ1-4GlcNAcβ1-6(GlcNAcβ1-3Galβ1-4GlcNAcβ1-3Galβ1-4GlcNAcβ1-3Galβ1-4GlcNAcβ1-3Galβ1-4GlcNAβ1-2)Manα1-6(GlcNAcβ1-3Galβ1-4GlcNAcβ1-3Galβ1-4GlcNAcβ1-3Galβ1-4GlcNAcβ1-3Galβ1-4GlcNAcβ1-2Manα1-3)Manβ1-4GlcNAcβ1-4(Fucα1-6)GlcNAcβ-Sp24	34	5
473	Fucα1-2Galβ1-3(Fucα1-4)GlcNAcβ1-2Manα1-6(Fucα1-2Galβ1-3(Fucα1-4)GlcNAcβ1-2Manα1-3)Manβ1-4GlcNAcβ1-4(Fucα1-6)GlcNAcβ1-4(Fucα1-6)GlcNAcβ-Sp19	33	12
388	GlcNAcβ1-2Manα1-6(GlcNAcβ1-4(GlcNAcβ1-2)Manα1-3)Manβ1-4GlcNAcβ1-4GlcNAc-Sp21	33	20
590	Galβ1-4GlcNAcβ1-3Galβ1-4GlcNAcβ1-6(Galβ1-3)GalNAcα-Sp14	33	15

138	Neu5Acα2-6(Galβ1-3)GlcNAcβ1-4Galβ1-4Glcβ-Sp10	33	17
115	Galα1-3Galβ1-4GlcNAcβ-Sp8	32	21
370	GalNAcα1-3(Fucα1-2)Galβ1-4GlcNAcβ1-2Manα1-6(GalNAcα1-3(Fucα1-2)Galβ1-4GlcNAcβ1-2Manα1-3)Manβ1-4GlcNAcβ1-4GlcNAcβ-Sp20	32	10
389	Fucα1-2Galβ1-3GalNAcα1-3(Fucα1-2)Galβ1-4Glcβ-Sp0	32	9
202	GlcAβ-Sp8	32	14
575	Galβ1-4GlcNAcβ1-3Galβ1-4GlcNAcβ1-2Manα1-6(Galβ1-4GlcNAcβ1-3Galβ1-4GlcNAcβ1-2Manα1-3)Manβ1-4GlcNAcβ1-4(Fucα1-6)GlcNAcβ-Sp24	32	13
582	Galβ1-4GlcNAcβ1-3Galβ1-4GlcNAcβ1-6(Galβ1-4GlcNAcβ1-3Galβ1-4GlcNAβ1-2)Manα1-6(Galβ1-4GlcNAcβ1-3Galβ1-4GlcNAcβ1-2Manα1-3)Manβ1-4GlcNAcβ1-4(Fucα1-6)GlcNAcβ-Sp24	31	14
411	Galβ1-3GalNAcβ1-4(Neu5Acα2-8Neu5Acα2-3)Galβ1-4Glcβ-Sp0	31	21
431	GlcNAcβ1-2Manα1-6(GlcNAcβ1-4)(GlcNAcβ1-2Manα1-3)Manβ1-4GlcNAcβ1-4GlcNAc-Sp21	31	22
136	Neu5Acα2-6(Galβ1-3)GalNAcα-Sp14	31	14
472	Fucα1-2Galβ1-4(Fucα1-3)GlcNAcβ1-2Manα1-6(Fucα1-2Galβ1-4(Fucα1-3)GlcNAcβ1-2Manα1-3)Manβ1-4GlcNAcβ1-4(Fucα1-6)GlcNAcβ-Sp24	30	9
83	GalNAcα1-3(Fucα1-2)Galβ1-4(Fucα1-3)GlcNAcβ-Sp0	30	22
455	Galα1-3(Fucα1-2)Galβ1-3GlcNAcβ1-2Manα1-6(Galα1-3(Fucα1-2)Galβ1-3GlcNAcβ1-2Manα1-3)Manβ1-4GlcNAcβ1-4(Fucα1-6)GlcNAcβ-Sp22	30	22
139	Galβ1-3GalNAcα-Sp8	30	28
104	Galα1-3(Fucα1-2)Galβ1-4(Fucα1-3)GlcNAcβ-Sp8	30	15
607	Neu5Acα2-3Galβ1-4GlcNAcβ1-3Galβ1-4GlcNAcβ1-3Galβ1-4GlcNAcβ1-2Manα1-6(Neu5Acα2-3Galβ1-4GlcNAcβ1-3Galβ1-4GlcNAcβ1-3Galβ1-4GlcNAcβ1-2Manα1-3)Manβ1-4GlcNAcβ1-4GlcNAcβ-Sp12	30	15
403	Galα1-4Galβ1-3GlcNAcβ1-2Manα1-6(Galα1-4Galβ1-3GlcNAcβ1-2Manα1-3)Manβ1-4GlcNAcβ1-4GlcNAcβ-Sp19	30	27
205	KDNα2-3Galβ1-3GlcNAcβ-Sp0	30	14
430	Fucα1-3GlcNAcβ1-6(Galβ1-4GlcNAcβ1-3)Galβ1-4Glc-Sp21	30	7
132	Galβ1-4GlcNAcβ1-6GalNAc-Sp14	29	9
264	Neu5Acα2-3Galβ1-4Glcβ-Sp8	29	7
454	GalNAcα1-3(Fucα1-2)Galβ1-4GlcNAcβ1-2Manα1-6(GalNAcα1-3(Fucα1-2)Galβ1-4GlcNAcβ1-2Manα1-3)Manβ1-4GlcNAcβ1-4(Fucα1-6)GlcNAcβ-Sp22	29	6
24	(3S)Galβ1-4(Fucα1-3)(6S)Glc-Sp0	29	17
82	GalNAcα1-3(Fucα1-2)Galβ1-3GlcNAcβ-Sp0	29	16
549	GlcNAcβ1-3Galβ1-4GlcNAcβ1-3Galβ1-4GlcNAcβ1-3Galβ1-4GlcNAcβ1-2Manα1-6(GlcNAcβ1-3Galβ1-4GlcNAcβ1-3Galβ1-4GlcNAcβ1-3Galβ1-4GlcNAcβ1-2Manα1-3)Manβ1-4GlcNAcβ1-4GlcNAcβ-Sp25	28	16
492	(3S)Galβ1-3(Fucα1-4)GlcNAcβ-Sp0	28	3
422	Fucα1-2Galβ1-3GlcNAcβ1-3GalNAc-Sp14	28	6

407	Galβ1-3GlcNAcα1-6Galβ1-4GlcNAcβ-Sp0	28	29
291	Galβ1-4(Fucα1-3)(6S)Glcβ-Sp0	28	16
244	Neu5Acα2-6(Neu5Acα2-3Galβ1-3)GalNAcα-Sp14	28	16
286	Neu5Gcα2-6Galβ1-4GlcNAcβ-Sp0	27	18
446	Fucα1-2Galβ1-4(Fucα1-3)GlcNAcβ1-2Manα1-6(Fucα1-2Galβ1-4(Fucα1-3)GlcNAcβ1-4(Fucα1-2Galβ1-4(Fucα1-3)GlcNAcβ1-2)Manα1-3)Manβ1-4GlcNAcβ1-4GlcNAcβ-Sp12	27	6
328	Neu5,9Ac2α2-3Galβ1-4GlcNAcβ-Sp0	27	24
425	Galα1-3Galβ1-3GlcNAcβ1-3GalNAc-Sp14	26	11
546	GlcNAcβ1-3Galβ1-4GlcNAcβ1-3Galβ1-4GlcNAcβ1-2Manα1-6(GlcNAcβ1-3Galβ1-4GlcNAcβ1-3Galβ1-4GlcNAcβ1-2Manα1-3)Manβ1-4GlcNAcβ1-4GlcNAcβ-Sp25	26	13
517	Galβ1-4(6P)GlcNAcβ-Sp0	26	23
81	Fucβ1-3GlcNAcβ-Sp8	26	5
591	Galβ1-4GlcNAcβ1-3Galβ1-4GlcNAcβ1-6(Galβ1-4GlcNAcβ1-3Galβ1-4GlcNAcβ1-3)GalNAcα-Sp14	26	11
414	GalNAcα1-3(Fucα1-2)Galβ1-4GlcNAcβ1-3GalNAcα-Sp14	26	35
125	Galβ1-2Galβ-Sp8	26	3
583	GlcNAcβ1-3Galβ1-4GlcNAcβ1-3Galβ1-4GlcNAcβ1-6(GlcNAcβ1-3Galβ1-4GlcNAcβ1-3Galβ1-4GlcNAβ1-2)Manα1-6(GlcNAcβ1-3Galβ1-4GlcNAcβ1-3Galβ1-4GlcNAcβ1-2Manα1-3)Manβ1-4GlcNAcβ1-4(Fucα1-6)GlcNAcβ-Sp24	25	9
586	Galβ1-4GlcNAcβ1-3Galβ1-4GlcNAcβ1-3Galβ1-4GlcNAcβ1-3Galβ1-4GlcNAcβ1-6(Galβ1-4GlcNAcβ1-3Galβ1-4GlcNAcβ1-3Galβ1-4GlcNAcβ1-3Galβ1-4GlcNAβ1-2)Manα1-6(Galβ1-4GlcNAcβ1-3Galβ1-4GlcNAcβ1-3Galβ1-4GlcNAcβ1-3Galβ1-4GlcNAcβ1-2Manα1-3)Manβ1-4GlcNAcβ1-4(Fucα1-6)GlcNAcβ-Sp24	25	9
257	Neu5Acα2-3Galβ1-4(Fucα1-3)GlcNAcβ1-3Galβ1-4GlcNAcβ-Sp8	25	23
392	GalNAcβ1-4(Neu5Acα2-3)Galβ1-4GlcNAcβ1-3GalNAcα-Sp14	25	18
448	Galβ1-4GlcNAcβ1-2Manα-Sp0	25	11
456	Neu5Acα2-6Galβ1-4GlcNAcβ1-6(Fucα1-2Galβ1-3GlcNAcβ1-3)Galβ1-4Glc-Sp21	24	10
457	GalNAcα1-3(Fucα1-2)Galβ1-3GlcNAcβ1-2Manα1-6(GalNAcα1-3(Fucα1-2)Galβ1-3GlcNAcβ1-2Manα1-3)Manβ1-4GlcNAcβ1-4(Fucα1-6)GlcNAcβ-Sp22	24	2
497	Fucα1-2(6S)Galβ1-3GlcNAcβ-Sp0	24	5
564	GlcNAcβ1-3Galβ1-4GlcNAcβ1-3Galβ1-4GlcNAcβ1-3Galβ1-4GlcNAcβ1-3Galβ1-4GlcNAcβ1-2Manα1-6(GlcNAcβ1-3Galβ1-4GlcNAcβ1-3Galβ1-4GlcNAcβ1-3Galβ1-4GlcNAcβ1-3Galβ1-4GlcNAcβ1-2Manα1-3)Manβ1-4GlcNAcβ1-4GlcNAcβ-Sp25	24	5
544	Fucα1-2Galβ1-4GlcNAcβ1-3Galβ1-4GlcNAcβ1-2Manα1-6(Fucα1-2Galβ1-4GlcNAcβ1-3Galβ1-4GlcNAcβ1-2Manα1-3)Manβ1-4GlcNAcβ1-4GlcNAcβ-Sp24	24	7
7	Fucα-Sp9	23	24
470	Glcα1-4Glcα1-4Glcα1-4Glcβ-Sp10	23	16

559	Galβ1-4GlcNAcβ1-3Galβ1-4GlcNAcβ1-6(Galβ1-4GlcNAcβ1-3Galβ1-4GlcNAcβ1-2)Manα1-6(Galβ1-4GlcNAcβ1-3Galβ1-4GlcNAcβ1-2Manα1-3)Manα1-4GlcNAcβ1-4GlcNAc-Sp24	23	31
574	GlcNAcβ1-3Galβ1-4GlcNAcβ1-2Manα1-6(GlcNAcβ1-3Galβ1-4GlcNAcβ1-2Manα1-3)Manβ1-4GlcNAcβ1-4(Fucα1-6)GlcNAcβ-Sp24	23	13
12	Galβ-Sp8	23	18
161	Galβ1-4GlcNAcβ1-3Galβ1-4(Fucα1-3)GlcNAcβ1-3Galβ1-4(Fucα1-3)GlcNAcβ-Sp0	23	4
42	(6S)Galβ1-4Glcβ-Sp0	23	6
399	Neu5Acα2-3Galβ1-3GlcNAcβ1-3GalNAcα-Sp14	23	24
438	Galβ1-4GlcNAcβ1-6(Galβ1-4GlcNAcβ1-2)Manα1-6(GlcNAcβ1-4)(Galβ1-4GlcNAcβ1-4(Galβ1-4GlcNAcβ1-2)Manα1-3)Manβ1-4GlcNAcβ1-4GlcNAc-Sp21	23	12
96	GalNAcβ1-3Galα1-4Galβ1-4GlcNAcβ-Sp0	23	7
568	Galβ1-4GlcNAcβ1-3Galβ1-4GlcNAcβ1-3Galβ1-4GlcNAcβ1-3Galβ1-4GlcNAcβ1-3Galβ1-4GlcNAcβ1-3Galβ1-4GlcNAcβ1-2Manα1-6(Galβ1-4GlcNAcβ1-3Galβ1-4GlcNAcβ1-3Galβ1-4GlcNAcβ1-3Galβ1-4GlcNAcβ1-3Galβ1-4GlcNAcβ1-3Galβ1-4GlcNAcβ1-2Manα1-3)Manβ1-4GlcNAcβ1-4GlcNAcβ-Sp25	23	11
580	GlcNAcβ1-3Galβ1-4GlcNAcβ1-3Galβ1-4GlcNAcβ1-3Galβ1-4GlcNAcβ1-3Galβ1-4GlcNAcβ1-2Manα1-6(GlcNAcβ1-3Galβ1-4GlcNAcβ1-3Galβ1-4GlcNAcβ1-3Galβ1-4GlcNAcβ1-3Galβ1-4GlcNAcβ1-2Manα1-3)Manβ1-4GlcNAcβ1-4(Fucα1-6)GlcNAcβ-Sp19	22	5
170	Galβ1-4GlcNAcβ-Sp23	22	17
284	Neu5Gcα2-3Galβ1-4Glcβ-Sp0	22	12
241	Neu5Acα2-3Galβ1-4(Neu5Acα2-3Galβ1-3)GlcNAcβ-Sp8	22	7
345	Neu5Acα2-6Galβ1-4GlcNAcβ1-2Manα1-6(Manα1-3)Manβ1-4GlcNAcβ1-4GlcNAc-Sp12	22	19
356	KDNα2-3Galβ1-4(Fucα1-3)GlcNAc-Sp0	22	3
584	Galβ1-4GlcNAcβ1-3Galβ1-4GlcNAcβ1-3Galβ1-4GlcNAcβ1-6(Galβ1-4GlcNAcβ1-3Galβ1-4GlcNAcβ1-3Galβ1-4GlcNAβ1-2)Manα1-6(Galβ1-4GlcNAcβ1-3Galβ1-4GlcNAcβ1-3Galβ1-4GlcNAcβ1-2Manα1-3)Manβ1-4GlcNAcβ1-4(Fucα1-6)GlcNAcβ-Sp24	22	7
118	Galα1-3Galβ-Sp8	22	6
400	Fucα1-2Galβ1-4GlcNAcβ1-3GalNAcα-Sp14	22	13
447	Galβ1-4(Fucα1-3)GlcNAcβ1-6GalNAc-Sp14	22	18
577	Galβ1-4GlcNAcβ1-3Galβ1-4GlcNAcβ1-3Galβ1-4GlcNAcβ1-2Manα1-6(Galβ1-4GlcNAcβ1-3Galβ1-4GlcNAcβ1-3Galβ1-4GlcNAcβ1-2Manα1-3)Manβ1-4GlcNAcβ1-4(Fucα1-6)GlcNAcβ-Sp24	22	24
288	Neu5Acα2-3Galβ1-4GlcNAcβ1-6(Galβ1-3)GalNAcα-Sp14	21	14
578	GlcNAcβ1-3Galβ1-4GlcNAcβ1-3Galβ1-4GlcNAcβ1-3Galβ1-4GlcNAcβ1-2Manα1-6(GlcNAcβ1-3Galβ1-4GlcNAcβ1-3Galβ1-4GlcNAcβ1-3Galβ1-4GlcNAcβ1-2Manα1-3)Manβ1-4GlcNAcβ1-4(Fucα1-6)GlcNAcβ-Sp24	21	14
490	Galβ1-3(Fucα1-4)GlcNAcβ1-6GalNAcα-Sp14	21	4
9	Neu5Acα-Sp8	21	10

417	Galα1-3(Fucα1-2)Galβ1-4(Fucα1-3)GlcNAcβ1-3GalNAc-Sp14	21	17
305	Galβ1-4GlcNAcβ1-6Galβ1-4GlcNAcβ-Sp0	21	9
601	Neu5Acα2-6Galβ1-4GlcNAcβ1-3Galβ1-4GlcNAcβ1-6(Galβ1-3)GalNAcα-Sp14	21	14
419	Galβ1-4(Fucα1-3)GlcNAcβ1-2Manα1-6(Galβ1-4(Fucα1-3)GlcNAcβ1-2Manα1-3)Manβ1-4GlcNAcβ1-4(Fucα1-6)GlcNAcβ-Sp22	21	10
520	Neu5Acα2-6Galβ1-4GlcNAcβ1-2Man-Sp0	21	8
374	Galα1-3(Fucα1-2)Galβ1-3GlcNAcβ1-2Manα1-6(Galα1-3(Fucα1-2)Galβ1-3GlcNAcβ1-2Manα1-3)Manβ1-4GlcNAcβ1-4GlcNAcβ-Sp20	20	8
240	Neu5Acα2-3Galβ1-3(Fucα1-4)GlcNAcβ1-3Galβ1-4(Fucα1-3)GlcNAcβ-Sp0	20	13
299	Neu5Acα2-3Galβ1-4(Fucα1-3)GlcNAcβ1-6(Galβ1-3)GalNAcα-Sp14	20	6
570	(3S)GlcAβ1-3Galβ1-4GlcNAcβ1-2Manα-Sp0	20	7
121	Galα1-4Galβ1-4GlcNAcβ-Sp8	20	16
122	Galα1-4Galβ1-4Glcβ-Sp0	20	3
280	Neu5Gcα2-3Galβ1-3(Fucα1-4)GlcNAcβ-Sp0	19	5
336	GalNAcα1-3(Fucα1-2)Galβ1-4GlcNAcβ1-3Galβ1-4GlcNAcβ1-3Galβ1-4GlcNAcβ-Sp0	19	8
307	GlcAβ1-3GlcNAcβ-Sp8	19	5
442	GalNAcβ1-6GalNAcβ-Sp8	19	13
203	GlcAβ1-3Galβ-Sp8	19	7
494	Fucα1-2Galβ1-4GlcNAcβ1-6GalNAcα-Sp14	19	0
543	Neu5Gcα2-3Galβ1-4GlcNAcβ1-3Galβ1-4GlcNAcβ1-2Manα1-6(Neu5Gcα2-3Galβ1-4GlcNAcβ1-3Galβ1-4GlcNAcβ1-2Manα1-3)Manβ1-4GlcNAcβ1-4GlcNAcβ-Sp24	19	8
153	Galβ1-4(Fucα1-3)GlcNAcβ1-3Galβ1-4(Fucα1-3)GlcNAcβ-Sp0	19	7
567	Galβ1-3GlcNAcβ1-6(Galβ1-3)GalNAc-Sp14	19	12
391	Galβ1-3GlcNAcβ1-3GalNAcα-Sp14	19	6
271	Neu5Acα2-6Galβ1-4GlcNAcβ1-3Galβ1-4GlcNAcβ-Sp0	19	12
281	Neu5Gcα2-3Galβ1-3GlcNAcβ-Sp0	18	10
579	Galβ1-4GlcNAcβ1-3Galβ1-4GlcNAcβ1-3Galβ1-4GlcNAcβ1-3Galβ1-4GlcNAcβ1-2Manα1-6(Galβ1-4GlcNAcβ1-3Galβ1-4GlcNAcβ1-3Galβ1-4GlcNAcβ1-3Galβ1-4GlcNAcβ1-2Manα1-3)Manβ1-4GlcNAcβ1-4(Fucα1-6)GlcNAcβ-Sp24	18	8
554	Neu5Gcα2-8Neu5Acα2-3Galβ1-4GlcNAc-Sp0	18	4
200	G-ol-Sp8	18	6
114	Galα1-3Galβ1-3GlcNAcβ-Sp0	18	11
443	(6S)Galβ1-3GlcNAcβ-Sp0	18	17
195	Glcα1-4Glcβ-Sp8	18	6

606	Neu5Acα2-6Galβ1-4GlcNAcβ1-3Galβ1-4GlcNAcβ1-3Galβ1-4GlcNAcβ1-2Manα1-6(Neu5Acα2-6Galβ1-4GlcNAcβ1-3Galβ1-4GlcNAcβ1-3Galβ1-4GlcNAcβ1-2Manα1-3)Manβ1-4GlcNAcβ1-4GlcNAcβ-Sp12	18	23
542	Galβ1-4GlcNAcβ1-3Galβ1-4GlcNAcβ1-2Manα1-6(Galβ1-4GlcNAcβ1-3Galβ1-4GlcNAcβ1-2Manα1-3)Manβ1-4GlcNAcβ1-4GlcNAcβ-Sp24	18	5
210	Manα1-6(Manα1-2Manα1-3)Manα1-6(Manα1-2Manα1-3)Manβ1-4GlcNAcβ1-4GlcNAcβ-Sp12	18	24
377	Neu5Acα2-6Galβ1-4GlcNAcβ1-3GalNAc-Sp14	18	7
588	Galβ1-4GlcNAcβ1-3Galβ1-4GlcNAcβ1-3Galβ1-4GlcNAcβ1-3Galβ1-4GlcNAcβ1-3Galβ1-4GlcNAcβ1-6(Galβ1-4GlcNAcβ1-3Galβ1-4GlcNAcβ1-3Galβ1-4GlcNAcβ1-3Galβ1-4GlcNAcβ1-3Galβ1-4GlcNAβ1-2)Manα1-6(Galβ1-4GlcNAcβ1-3Galβ1-4GlcNAcβ1-3Galβ1-4GlcNAcβ1-3Galβ1-4GlcNAcβ1-3Galβ1-4GlcNAcβ1-2Manα1-3)Manβ1-4GlcNAcβ1-4(Fucα1-6)GlcNAcβ-Sp24	18	9
223	Neu5Acα2-3Galβ1-3GalNAcα-Sp8	18	13
143	Galβ1-3GalNAcβ1-3Galα1-4Galβ1-4Glcβ-Sp0	18	18
376	Neu5Acα2-3Galβ1-4GlcNAcβ1-3GalNAc-Sp14	18	7
318	Neu5Acα2-6Galβ1-4GlcNAcβ1-2Manα1-6(Neu5Acα2-3Galβ1-4GlcNAcβ1-2Manα1-3)Manβ1-4GlcNAcβ1-4GlcNAcβ-Sp12	18	11
384	Galβ1-4GlcNAcβ1-6(Fucα1-4(Fucα1-2Galβ1-3)GlcNAcβ1-3)Galβ1-4Glc-Sp21	18	12
16	GlcNAcβ-Sp0	18	10
206	KDNα2-3Galβ1-4GlcNAcβ-Sp0	18	12
50	Manα1-6(Manα1-3)Manβ1-4GlcNAcβ1-4GlcNAcβ-Sp12	18	7
213	Manα1-6(Manα1-3)Manα-Sp9	17	9
581	Galβ1-4GlcNAcβ1-3Galβ1-4GlcNAcβ1-3Galβ1-4GlcNAcβ1-3Galβ1-4GlcNAcβ1-3Galβ1-4GlcNAcβ1-2Manα1-6(Galβ1-4GlcNAcβ1-3Galβ1-4GlcNAcβ1-3Galβ1-4GlcNAcβ1-3Galβ1-4GlcNAcβ1-3Galβ1-4GlcNAcβ1-2Manα1-3)Manβ1-4GlcNAcβ1-4(Fucα1-6)GlcNAcβ-Sp19	17	3
576	GlcNAcβ1-3Galβ1-4GlcNAcβ1-3Galβ1-4GlcNAcβ1-2Manα1-6(GlcNAcβ1-3Galβ1-4GlcNAcβ1-3Galβ1-4GlcNAcβ1-2Manα1-3)Manβ1-4GlcNAcβ1-4(Fucα1-6)GlcNAcβ-Sp24	17	4
326	Neu5Acα2-3Galβ1-4GlcNAcβ1-2Manα1-6(Neu5Acα2-6Galβ1-4GlcNAcβ1-2Manα1-3)Manβ1-4GlcNAcβ1-4GlcNAcβ-Sp12	17	10
6	Fucα-Sp8	17	5
145	Galβ1-3GalNAcβ1-4Galβ1-4Glcβ-Sp8	17	16
439	Galβ1-4Galβ-Sp10	17	7
529	Neu5Acα2-3Galβ1-3GlcNAcβ1-4Galβ1-4Glcβ-Sp0	17	20
106	Galα1-3(Fucα1-2)Galβ1-4Glcβ-Sp0	17	11
589	Galβ1-4GlcNAcβ1-3Galβ1-4GlcNAcβ1-3GalNAcα-Sp14	17	5
504	(3S)GalNAcβ1-4(Fucα1-3)GlcNAcβ-Sp8	17	8

482	Neu5Acα2-6Galβ1-4GlcNAcβ1-2Manα1-6(Neu5Acα2-6Galβ1-4GlcNAcβ1-2Manα1-3)Manβ1-4GlcNAcβ1-4(Fucα1-6)GlcNAcβ-Sp24	17	9
11	Neu5Acβ-Sp8	17	6
231	GalNAcβ1-4(Neu5Acα2-3)Galβ1-4GlcNAcβ-Sp0	16	16
462	Neu5Acα2-3Galβ1-4GlcNAcβ1-6(Neu5Acα2-3Galβ1-4GlcNAcβ1-2)Manα1-6(GlcNAcβ1-4)(Neu5Acα2-3Galβ1-4GlcNAcβ1-4(Neu5Acα2-3Galβ1-4GlcNAcβ1-2)Manα1-3)Manβ1-4GlcNAcβ1-4GlcNAcβ-Sp21	16	8
35	(3S)Galβ1-4(6S)GlcNAcβ-Sp8	16	3
58	Fucα1-2Galβ1-3GalNAcβ1-3Galα-Sp9	16	13
550	Galβ1-4GlcNAcβ1-3Galβ1-4GlcNAcβ1-3Galβ1-4GlcNAcβ1-3Galβ1-4GlcNAcβ1-2Manα1-6(Galβ1-4GlcNAcβ1-3Galβ1-4GlcNAcβ1-3Galβ1-4GlcNAcβ1-3Galβ1-4GlcNAcβ1-2Manα1-3)Manβ1-4GlcNAcβ1-4GlcNAcβ-Sp25	16	16
109	Galα1-4(Galα1-3)Galβ1-4GlcNAcβ-Sp8	16	12
498	Galα1-3(Fucα1-2)Galβ1-4GlcNAcβ1-6GalNAcα-Sp14	16	14
227	Neu5Acα2-8Neu5Acα2-8Neu5Acα2-3Galβ1-4Glcβ-Sp0	16	9
420	Fucα1-2Galβ1-4GlcNAcβ1-2Manα1-6(Fucα1-2Galβ1-4GlcNAcβ1-2Manα1-3)Manβ1-4GlcNAcβ1-4(Fucα1-6)GlcNAcβ-Sp22	16	9
127	Galβ1-3GlcNAcβ1-3Galβ1-4(Fucα1-3)GlcNAcβ-Sp0	16	9
209	Manα1-2Manα1-3Manα-Sp9	16	15
59	Fucα1-2Galβ1-3GalNAcβ1-3Galα1-4Galβ1-4Glcβ-Sp9	16	9
92	GalNAcα1-3Galβ-Sp8	16	8
226	GalNAcβ1-4(Neu5Acα2-8Neu5Acα2-8Neu5Acα2-3)Galβ1-4Glcβ-Sp0	16	12
489	Galα1-3Galβ1-3GlcNAcβ1-6GalNAcα-Sp14	16	9
184	GlcNAcβ1-3Galβ1-4GlcNAcβ1-3Galβ1-4GlcNAcβ-Sp0	16	5
347	Neu5Acα2-6Galβ1-4GlcNAcβ1-2Manα1-6Manβ1-4GlcNAcβ1-4GlcNAc-Sp12	16	3
289	Galβ1-3GlcNAcβ1-3Galβ1-3GlcNAcβ-Sp0	15	9
20	Galβ1-4GlcNAcβ1-6(Galβ1-4GlcNAcβ1-3)GalNAc-Sp14	15	7
495	Galα1-3Galβ1-4GlcNAcβ1-6GalNAcα-Sp14	15	10
102	Galα1-3(Fucα1-2)Galβ1-3GlcNAcβ-Sp8	15	11
424	GalNAcα1-3(Fucα1-2)Galβ1-3GlcNAcβ1-3GalNAc-Sp14	15	7
344	GlcNAcα1-4Galβ1-3GalNAc-Sp14	15	14
562	GalNAcβ1-3GlcNAcβ-Sp0	15	14
525	Galα1-3(Fucα1-2)Galβ1-3GlcNAcβ1-6GalNAc-Sp14	15	6
88	GlcNAcβ1-3Galβ1-3GalNAcα-Sp8	15	2
510	Galβ1-3(6S)GlcNAcβ-Sp8	15	5
290	Galβ1-4(Fucα1-3)(6S)GlcNAcβ-Sp0	15	5
348	Neu5Acα2-6Galβ1-4GlcNAcβ1-2Manα1-3Manβ1-4GlcNAcβ1-4GlcNAc-Sp12	14	10

603	Neu5Acα2-3Galβ1-4GlcNAcβ1-3Galβ1-4GlcNAcβ1-2Manα1-6(Neu5Acα2-3Galβ1-4GlcNAcβ1-3Galβ1-4GlcNAcβ1-2Manα1-3)Manβ1-4GlcNAcβ1-4GlcNAcβ-Sp12	14	13
238	Neu5Acα2-3Galβ1-3(6S)GlcNAc-Sp8	14	9
551	Galβ1-3GlcNAcβ1-3Galβ1-4GlcNAcβ1-2Manα1-6(Galβ1-3GlcNAcβ1-3Galβ1-4GlcNAcβ1-2Manα1-3)Manβ1-4GlcNAcβ1-4GlcNAc-Sp25	14	6
445	Fucα1-2Galβ1-4 GlcNAcβ1-2Manα1-6(Fucα1-2Galβ1-4GlcNAcβ1-2(Fucα1-2Galβ1-4GlcNAcβ1-4)Manα1-3)Manβ1-4GlcNAcβ1-4GlcNAcβ-Sp12	14	6
314	Manα1-6(Manα1-3)Manα1-6(Manα1-3)Manβ-Sp10	14	7
123	Galα1-4GlcNAcβ-Sp8	14	11
317	Neu5Acα2-3Galβ1-4GlcNAcβ1-6(Neu5Acα2-3Galβ1-3)GalNAcα-Sp14	14	9
36	(3S)Galβ1-4GlcNAcβ-Sp0	14	4
151	Galβ1-4(Fucα1-3)GlcNAcβ-Sp0	14	6
602	Neu5Acα2-6Galβ1-4GlcNAcβ1-6(Galβ1-3)GalNAcα-Sp14	14	4
330	Neu5Acα2-6Galβ1-4GlcNAcβ1-3Galβ1-3GlcNAcβ-Sp0	14	3
528	GalNAcβ1-4GlcNAcβ1-2Manα-Sp0	14	4
382	Galβ1-3GlcNAcβ1-3Galβ1-4GlcNAcβ1-6(Galβ1-3GlcNAcβ1-3)Galβ1-4Glcβ-Sp0	14	4
124	Galα1-6Glcβ-Sp8	13	4
477	Neu5Acα2-6Galβ1-4GlcNAcβ1-6(Galβ1-3GlcNAcβ1-3)Galβ1-4Glcβ-Sp21	13	2
67	Fucα1-2Galβ1-3GlcNAcβ-Sp0	13	5
540	GlcNAcβ1-3Galβ1-4GlcNAcβ1-2Manα1-6(GlcNAcβ1-3Galβ1-4GlcNAcβ1-2Manα1-3)Manβ1-4GlcNAcβ1-4GlcNAcβ-Sp25	13	6
283	Neu5Gcα2-3Galβ1-4GlcNAcβ-Sp0	13	4
3	Manα-Sp8	13	7
158	Galβ1-4GalNAcβ1-3(Fucα1-2)Galβ1-4GlcNAcβ-Sp8	13	9
221	Fucα1-2Galβ1-4(6S)GlcNAcβ-Sp8	13	3
219	(3S)Galβ1-4(Fucα1-3)(6S)GlcNAcβ-Sp8	13	9
496	Galβ1-4(Fucα1-3)GlcNAcβ1-2Manα-Sp0	13	7
327	Galβ1-4(Fucα1-3)GlcNAcβ1-2Manα1-6(Galβ1-4(Fucα1-3)GlcNAcβ1-2Manα1-3)Manβ1-4GlcNAcβ1-4GlcNAcβ-Sp20	13	4
79	Fucα1-3GlcNAcβ-Sp8	13	9
48	Neu5,9Ac$_2$α-Sp8	13	8
585	GlcNAcβ1-3Galβ1-4GlcNAcβ1-3Galβ1-4GlcNAcβ1-3Galβ1-4GlcNAcβ1-6(GlcNAcβ1-3Galβ1-4GlcNAcβ1-3Galβ1-4GlcNAcβ1-3Galβ1-4GlcNAβ1-2)Manα1-6(GlcNAcβ1-3Galβ1-4GlcNAcβ1-3Galβ1-4GlcNAcβ1-3Galβ1-4GlcNAcβ1-2Manα1-3)Manβ1-4GlcNAcβ1-4(Fucα1-6)GlcNAcβ-Sp24	13	2
2	Glcα-Sp8	13	16

565	Galβ1-4GlcNAcβ1-3Galβ1-4GlcNAcβ1-3Galβ1-4GlcNAcβ1-3Galβ1-4GlcNAcβ1-3Galβ1-4GlcNAcβ1-2Manα1-6(Galβ1-4GlcNAcβ1-3Galβ1-4GlcNAcβ1-3Galβ1-4GlcNAcβ1-3Galβ1-4GlcNAcβ1-3Galβ1-4GlcNAcβ1-2Manα1-3)Manβ1-4GlcNAcβ1-4GlcNAcβ-Sp25	13	11
466	Neu5Acα2-6Galβ1-4GlcNAcβ1-6(Neu5Acα2-6Galβ1-4GlcNAcβ1-2)Manα1-6(GlcNAcβ1-4)(Neu5Acα2-6Galβ1-4GlcNAcβ1-4(Neu5Acα2-6Galβ1-4GlcNAcβ1-2)Manα1-3)Manβ1-4GlcNAcβ1-4GlcNAcβ-Sp21	13	7
599	Galβ1-4GlcNAcβ1-3Galβ1-3GalNAcα-Sp14	13	12
214	Manα1-2Manα1-2Manα1-6(Manα1-3)Manα-Sp9	12	5
128	Galβ1-3(Fucα1-4)GlcNAc-Sp0	12	12
279	Neu5Acb2-6Galβ1-4GlcNAcβ-Sp8	12	12
282	Neu5Gcα2-3Galβ1-4(Fucα1-3)GlcNAcβ-Sp0	12	12
134	GlcNAcβ1-6(Galβ1-3)GalNAcα-Sp14	12	8
133	GlcNAcβ1-6(Galβ1-3)GalNAcα-Sp8	12	4
43	(6S)Galβ1-4Glcβ-Sp8	12	6
593	GlcNAcβ1-3Galβ1-4GlcNAcβ1-3GalNAcα-Sp14	12	7
609	GlcNAcβ1-3Fucα-Sp21	12	5
514	GalNAcβ1-4(6S)GlcNAc-Sp8	12	7
140	Galβ1-3GalNAcα-Sp14	12	12
536	GalNAcα1-3(Fucα1-2)Galβ1-3GalNAcβ1-3Galα1-4Galβ1-4Glc-Sp21	12	4
433	GlcNAcβ1-6(GlcNAcβ1-2)Manα1-6(GlcNAcβ1-4)(GlcNAcβ1-2Manα1-3)Manβ1-4GlcNAcβ1-4GlcNAc-Sp21	12	9
63	Fucα1-2Galβ1-3GalNAcβ1-4(Neu5Acα2-3)Galβ1-4Glcβ-Sp0	12	7
22	6S(3S)Galβ1-4(6S)GlcNAcβ-Sp0	12	3
548	Galβ1-4GlcNAcβ1-3Galβ1-4GlcNAcβ1-3Galβ1-4GlcNAcβ1-2Manα1-6(Galβ1-4GlcNAcβ1-3Galβ1-4GlcNAcβ1-3Galβ1-4GlcNAcβ1-2Manα1-3)Manβ1-4GlcNAcβ1-4GlcNAcβ-Sp24	12	7
194	GlcNAcβ1-6Galβ1-4GlcNAcβ-Sp8	12	9
481	Neu5Acα2-6Galβ1-4 GlcNAcβ1-6(Neu5Acα2-6Galβ1-4GlcNAcβ1-3)GalNAcα-Sp14	12	7
18	GlcN(Gc)β-Sp8	12	11
33	(3S)Galβ1-4(Fucα1-3)GlcNAc-Sp8	12	6
23	6S(3S)Galβ1-4GlcNAcβ-Sp0	12	7
183	GlcNAcβ1-3Galβ1-4GlcNAcβ-Sp8	12	5
277	Galβ1-3(Fucα1-4)GlcNAcβ1-3Galβ1-3(Fucα1-4)GlcNAcβ-Sp0	12	11
167	Galβ1-4GlcNAcβ1-6(Galβ1-3)GalNAc-Sp14	12	5
483	Neu5Acα2-3Galβ1-4GlcNAcβ1-2Manα1-6(Neu5Acα2-3Galβ1-4GlcNAcβ1-2Manα1-3)Manβ1-4GlcNAcβ1-4(Fucα1-6)GlcNAcβ-Sp24	12	6
311	GlcNAcβ1-4GlcNAcβ-Sp12	12	9
258	Neu5Acα2-3Galβ1-4GlcNAcβ1-3Galβ1-4GlcNAcβ1-3Galβ1-4GlcNAcβ-Sp0	12	10

38	(3S)Galβ-Sp8	12	20
595	GlcNAcβ1-3Galβ1-4GlcNAcβ1-6(GlcNAcβ1-3Galβ1-4GlcNAcβ1-3)GalNAcα-Sp14	12	5
172	Galβ1-4Glcβ-Sp8	12	2
315	Manα1-2Manα1-6(Manα1-3)Manα1-6(Manα1-2Manα1-2Manα1-3)Manα-Sp9	11	4
185	GlcNAcβ1-3Galβ1-4Glcβ-Sp0	11	11
297	(6S)Galβ1-4(6S)GlcNAcβ-Sp0	11	13
390	Fucα1-2Galβ1-3GalNAcα1-3(Fucα1-2)Galβ1-4GlcNAcβ-Sp0	11	8
566	GlcNAβ1-3Galβ1-3GalNAc-Sp14	11	7
26	(3S)Galβ1-4(6S)Glcβ-Sp0	11	12
396	Neu5Acα2-3Galβ1-3GlcNAcβ1-2Manα1-6(Neu5Acα2-3Galβ1-3GlcNAcβ1-2Manα1-3)Manβ1-4GlcNAcβ1-4GlcNAc-Sp19	11	4
177	GlcNAcβ1-6(GlcNAcβ1-3)GalNAcα-Sp14	11	5
491	Neu5Acα2-3Galβ1-3GlcNAcβ1-6GalNAcα-Sp14	11	11
71	Fucα1-2Galβ1-4(Fucα1-3)GlcNAcβ-Sp0	11	4
66	Fucα1-2Galβ1-3GlcNAcβ1-3Galβ1-4Glcβ-Sp10	11	13
516	(4S)GalNAcβ-Sp10	11	2
188	GlcNAcβ1-4Galβ1-4GlcNAcβ-Sp8	11	5
91	GalNAcα1-3GalNAcβ-Sp8	11	4
141	Galβ1-3GalNAcα-Sp16	11	6
429	Galβ1-4GlcNAcβ1-6(Fucα1-2Galβ1-3GlcNAcβ1-3)Galβ1-4Glc-Sp21	11	10
131	Galβ1-4GlcNAcβ1-6GalNAcα-Sp8	11	6
329	Neu5,9Ac2α2-3Galβ1-3GlcNAcβ-Sp0	11	12
70	Fucα1-2Galβ1-4(Fucα1-3)GlcNAcβ1-3Galβ1-4(Fucα1-3)GlcNAcβ1-3Galβ1-4(Fucα1-3)GlcNAcβ-Sp0	11	5
116	Galα1-3Galβ1-4Glcβ-Sp0	11	5
597	Neu5Acα2-6Galβ1-4GlcNAcβ1-3Galβ1-4GlcNAcβ1-3GalNAcα-Sp14	10	6
386	Galβ1-3GlcNAcβ1-3Galβ1-4(Fucα1-3)GlcNAcβ1-6(Galβ1-3GlcNAcβ1-3)Galβ1-4Glc-Sp21	10	12
309	GlcNAcβ1-3Man-Sp10	10	13
39	(6S)(4S)Galβ1-4GlcNAcβ-Sp0	10	10
368	Galβ1-4(Fucα1-3)GlcNAcβ1-6(Fucα1-2Galβ1-4GlcNAcβ1-3)Galβ1-4Glc-Sp21	10	5
21	GlcNAcβ1-6(GlcNAcβ1-4)(GlcNAcβ1-3)GlcNAc-Sp8	10	12
300	Galβ1-3Galβ1-4GlcNAcβ-Sp8	10	8
460	Neu5Acα2-3Galβ1-4GlcNAcβ1-4Manα1-6(GlcNAcβ1-4)(Neu5Acα2-3Galβ1-4GlcNAcβ1-4(Neu5Acα2-3Galβ1-4GlcNAcβ1-2)Manα1-3)Manβ1-4GlcNAcβ1-4GlcNAcβ-Sp21	10	7
236	Neu5Acα2-3GalNAcα-Sp8	10	12
515	(3S)GalNAcβ1-4GlcNAc-Sp8	10	6

212	Manα1-2Manα1-6(Manα1-2Manα1-3)Manα1-6(Manα1-2Manα1-2Manα1-3)Manβ1-4GlcNAcβ1-4GlcNAcβ-Sp12	10	12
373	GalNAcα1-3(Fucα1-2)Galβ1-3GlcNAcβ1-2Manα1-6(GalNAcα1-3(Fucα1-2)Galβ1-3GlcNAcβ1-2Manα1-3)Manβ1-4GlcNAcβ1-4GlcNAcβ-Sp20	10	9
45	(6S)Galβ1-4(6S)Glcβ-Sp8	10	6
87	GalNAcα1-3(Fucα1-2)Galβ1-4Glcβ-Sp0	10	10
187	GlcNAcβ1-6(GlcNAcβ1-4)GalNAcα-Sp8	10	5
19	Galβ1-4GlcNAcβ1-6(Galβ1-4GlcNAcβ1-3)GalNAcα-Sp8	10	4
94	GalNAcβ1-3GalNAcα-Sp8	10	2
488	Galβ1-3GlcNAcβ1-6GalNAcα-Sp14	10	8
432	GlcNAcβ1-2Manα1-6(GlcNAcβ1-4)(GlcNAcβ1-4(GlcNAcβ1-2)Manα1-3)Manβ1-4GlcNAcβ1-4GlcNAc-Sp21	10	1
255	Neu5Acα2-3Galβ1-4(Fucα1-3)GlcNAcβ-Sp8	10	4
538	Galβ1-3GalNAcβ1-3Gal-Sp21	10	6
135	Neu5Acα2-6(Galβ1-3)GalNAcα-Sp8	10	6
323	Neu5Gcb2-6Galβ1-4GlcNAc-Sp8	10	5
17	GlcNAcβ-Sp8	10	15
61	Fucα1-2Galβ1-3GalNAcα-Sp8	10	3
285	Neu5Gcα2-6GalNAcα-Sp0	10	6
535	GlcNAcβ1-3Galβ1-4GlcNAcβ1-6(GlcNAcβ1-3)Galβ1-4GlcNAc-Sp0	9	6
278	Neu5Acb2-6GalNAcα-Sp8	9	7
37	(3S)Galβ1-4GlcNAcβ-Sp8	9	10
406	Galβ1-3GlcNAcβ1-6Galβ1-4GlcNAcβ-Sp0	9	9
484	Manα1-6(Manα1-3)Manβ1-4GlcNAcβ1-4(Fucα1-6)GlcNAcβ-Sp19	9	5
89	GalNAcα1-3(Fucα1-2)Galβ-Sp8	9	2
251	Neu5Acα2-3Galβ1-4(6S)GlcNAcβ-Sp8	9	11
72	Fucα1-2Galβ1-4(Fucα1-3)GlcNAcβ-Sp8	9	8
218	Neu5Acα2-3Galβ1-4GlcNAcβ1-3Galβ1-4(Fucα1-3)GlcNAcβ-Sp0	9	6
509	Galβ1-3GlcNAcα1-3Galβ1-4GlcNAcβ-Sp8	9	7
25	(3S)Galβ1-4Glcβ-Sp8	9	2
29	(3S)Galβ1-3GalNAcα-Sp8	9	8
247	Neu5Acα2-3Galβ1-3GlcNAcβ1-3Galβ1-4GlcNAcβ-Sp0	9	6
239	Neu5Acα2-3Galβ1-3(Fucα1-4)GlcNAcβ-Sp8	9	6
503	GalNAcβ1-4(Fucα1-3)(6S)GlcNAcβ-Sp8	9	6
150	Galβ1-3GlcNAcβ-Sp8	9	5
511	(6S)(4S)GalNAcβ1-4GlcNAc-Sp8	9	6

539	GlcNAcβ1-3Galβ1-4GlcNAcβ1-2Manα1-6(GlcNAcβ1-3Galβ1-4GlcNAcβ1-2Manα1-3)Manβ1-4GlcNAcβ1-4GlcNAcβ-Sp12	9	3
263	Neu5Acα2-3Galβ1-4Glcβ-Sp0	9	3
343	GlcNAcα1-4Galβ1-4GlcNAcβ1-3Galβ1-4GlcNAcβ-Sp0	9	13
201	GlcAα-Sp8	9	8
608	Neu5Acα2-6Galβ1-4GlcNAcβ1-3Galβ1-4GlcNAcβ1-2Manα1-6(Neu5Acα2-6Galβ1-4GlcNAcβ1-3Galβ1-4GlcNAcβ1-2Manα1-3)Manβ1-4GlcNAcβ1-4GlcNAcβ-Sp12	9	6
465	Neu5Acα2-6Galβ1-4GlcNAcβ1-6(Neu5Acα2-6Galβ1-4GlcNAcβ1-2)Manα1-6(GlcNAcβ1-4)(Neu5Acα2-6Galβ1-4GlcNAcβ1-2Manα1-3)Manβ1-4GlcNAcβ1-4GlcNAcβ-Sp21	9	2
27	(3S)Galβ1-4(6S)Glcβ-Sp8	9	6
193	GlcNAcβ1-6GalNAcα-Sp14	9	5
47	(6S)GlcNAcβ-Sp8	9	15
316	Manα1-2Manα1-6(Manα1-2Manα1-3)Manα1-6(Manα1-2Manα1-2Manα1-3)Manα-Sp9	9	8
103	Galα1-3(Fucα1-2)Galβ1-4(Fucα1-3)GlcNAcβ-Sp0	9	8
57	Neu5Acα2-6Galβ1-4GlcNAcβ1-2Manα1-6(Neu5Acα2-6Galβ1-4GlcNAcβ1-2Manα1-3)Manβ1-4GlcNAcβ1-4GlcNAcβ-Sp24	9	9
192	GlcNAcβ1-6GalNAcα-Sp8	9	6
5	GalNAcα-Sp15	9	5
333	Galα1-4Galβ1-4GlcNAcβ1-3Galβ1-4Glcβ-Sp0	9	6
233	GalNAcβ1-4(Neu5Acα2-3)Galβ1-4Glcβ-Sp0	9	7
208	Manα1-2Manα1-6(Manα1-2Manα1-3)Manα-Sp9	9	13
378	Neu5Acα2-3Galβ1-4(Fucα1-3)GlcNAcβ1-3GalNAcα-Sp14	9	6
14	Manβ-Sp8	9	10
380	Galβ1-3GalNAcα1-3(Fucα1-2)Galβ1-4Glc-Sp0	9	8
459	Neu5Acα2-3Galβ1-4GlcNAcβ1-2Manα1-6(GlcNAcβ1-4)(Neu5Acα2-3Galβ1-4GlcNAcβ1-2Manα1-3)Manβ1-4GlcNAcβ1-4GlcNAcβ-Sp21	9	4
230	Neu5Acα2-3(6S)Galβ1-4(Fucα1-3)GlcNAcβ-Sp8	9	12
512	(6S)GalNAcβ1-4GlcNAc-Sp8	9	10
556	Neu5Gcα2-8Neu5Gcα2-6Galβ1-4GlcNAc-Sp0	8	4
534	Neu5Acα2-3Galβ1-4(Fucα1-3)GlcNAcβ1-2Manα-Sp0	8	5
331	Neu5Acα2-3Galβ1-3(Fucα1-4)GlcNAcβ1-3Galβ1-3(Fucα1-4)GlcNAcβ-Sp0	8	5
513	(3S)GalNAcβ1-4(3S)GlcNAc-Sp8	8	3
295	Neu5Acα2-3Galβ1-4GlcNAcβ1-3Galβ1-3GlcNAcβ-Sp0	8	7
254	Neu5Acα2-3Galβ1-4(Fucα1-3)GlcNAcβ-Sp0	8	6
592	Neu5Acα2-3Galβ1-4GlcNAcβ1-3Galβ1-4GlcNAcβ1-3GalNAcα-Sp14	8	10

261	Neu5Acα2-3Galβ1-4GlcNAcβ1-3Galβ1-4GlcNAcβ-Sp0	8	6
359	KDNα2-3Galβ1-3GalNAcα-Sp14	8	4
246	Neu5Acα2-3Galβ1-3GalNAcβ1-3Galα1-4Galβ1-4Glcβ-Sp0	8	4
149	Galβ1-3GlcNAcβ-Sp0	8	2
248	Fucα1-2(6S)Galβ1-4Glcβ-Sp0	8	5
292	Galβ1-4(Fucα1-3)GlcNAcβ1-3Galβ1-3(Fucα1-4)GlcNAcβ-Sp0	8	8
31	(3S)Galβ1-3GlcNAcβ-Sp8	8	6
216	Manα1-6(Manα1-3)Manα1-6(Manα1-3)Manβ1-4GlcNAcβ1-4GlcNAcβ-Sp12	8	2
1	Galα-Sp8	8	8
32	(3S)Galβ1-4(Fucα1-3)GlcNAc-Sp0	8	8
176	GlcNAcβ1-6(GlcNAcβ1-3)GalNAcα-Sp8	8	5
179	GlcNAcβ1-3GalNAcα-Sp8	8	4
159	Galβ1-4GlcNAcβ1-3GalNAcα-Sp8	8	6
165	Galβ1-4GlcNAcβ1-3Galβ1-4Glcβ-Sp8	8	12
8	Rhaα-Sp8	8	4
338	GlcNAcα1-4Galβ1-4GlcNAcβ1-3Galβ1-4GlcNAcβ1-3Galβ1-4GlcNAcβ-Sp0	8	10
100	Galα1-2Galβ-Sp8	8	5
117	Galα1-3Galβ1-4Glc-Sp10	8	5
303	GlcNAcβ1-6(Galβ1-4GlcNAcβ1-3)Galβ1-4GlcNAc-Sp0	8	5
148	Galβ1-3GlcNAcβ1-3Galβ1-4Glcβ-Sp10	8	8
405	Galα1-3Galβ1-4GlcNAcβ1-3GalNAcα-Sp14	8	6
191	GlcNAcβ1-4GlcNAcβ1-4GlcNAcβ-Sp8	8	7
275	Neu5Acα2-8Neu5Acα-Sp8	8	8
60	Fucα1-2Galβ1-3(Fucα1-4)GlcNAcβ-Sp8	8	10
250	Neu5Acα2-3Galβ1-3GlcNAcβ-Sp8	7	3
110	Galα1-3GalNAcα-Sp8	7	6
463	Neu5Acα2-6Galβ1-4GlcNAcβ1-2Manα1-6(GlcNAcβ1-4)(Neu5Acα2-6Galβ1-4GlcNAcβ1-2Manα1-3)Manβ1-4GlcNAcβ1-4GlcNAcβ-Sp21	7	5
310	GlcNAcβ1-4GlcNAcβ-Sp10	7	4
180	GlcNAcβ1-3GalNAcα-Sp14	7	5
113	Galα1-3Galβ1-4(Fucα1-3)GlcNAcβ-Sp8	7	7
232	GalNAcβ1-4(Neu5Acα2-3)Galβ1-4GlcNAcβ-Sp8	7	4
120	Galα1-4Galβ1-4GlcNAcβ-Sp0	7	6
228	GalNAcβ1-4(Neu5Acα2-8Neu5Acα2-3)Galβ1-4Glcβ-Sp0	7	4
505	Fucα1-2Galβ1-3GlcNAcβ1-6(Fucα1-2Galβ1-3GlcNAcβ1-3)GalNAcα-Sp14	7	3

46	Neu5Acα2-3(6S)Galβ1-4GlcNAcβ-Sp8	7	7
487	Neu5Acα2-6Galβ1-4GlcNAcβ1-6(Fucα1-2Galβ1-4(Fucα1-3)GlcNAcβ1-3)Galβ1-4Glc-Sp21	7	5
237	Neu5Acα2-3GalNAcβ1-4GlcNAcβ-Sp0	7	3
147	Galβ1-3GlcNAcβ1-3Galβ1-4GlcNAcβ-Sp0	7	5
594	GlcNAcβ1-3Galβ1-4GlcNAcβ1-6(Galβ1-3)GalNAcα-Sp14	7	13
144	Galβ1-3GalNAcβ1-4(Neu5Acα2-3)Galβ1-4Glcβ-Sp0	7	5
253	Neu5Acα2-3Galβ1-4(Fucα1-3)GlcNAcβ1-3Galβ1-4(Fucα1-3)GlcNAcβ1-3Galβ1-4(Fucα1-3)GlcNAcβ-Sp0	7	11
408	GalNAcβ1-3Galα1-6Galβ1-4Glcβ-Sp8	7	4
80	Fucα1-4GlcNAcβ-Sp8	7	7
199	Glcβ1-6Glcβ-Sp8	7	12
157	Galβ1-4GalNAcα1-3(Fucα1-2)Galβ1-4GlcNAcβ-Sp8	7	5
561	GlcNAcβ1-3Galβ1-4GlcNAcβ1-6(GlcNAcβ1-3Galβ1-3)GalNAcα-Sp14	7	4
321	Neu5Acα2-8Neu5Acβ-Sp17	7	3
304	Galβ1-4GlcNAcα1-6Galβ1-4GlcNAcβ-Sp0	7	10
111	Galα1-3GalNAcα-Sp16	7	7
74	Fucα1-2Galβ1-4GlcNAcβ1-3Galβ1-4GlcNAcβ1-3Galβ1-4GlcNAcβ-Sp0	7	4
287	Neu5Gcα-Sp8	7	5
222	Fucα1-2(6S)Galβ1-4(6S)Glcβ-Sp0	7	5
220	Fucα1-2(6S)Galβ1-4GlcNAcβ-Sp0	7	6
322	Neu5Acα2-8Neu5Acα2-8Neu5Acβ-Sp8	7	6
262	Fucα1-2Galβ1-4(6S)Glcβ-Sp0	7	2
93	GalNAcα1-4(Fucα1-2)Galβ1-4GlcNAcβ-Sp8	7	2
573	Neu5Acα2-8Neu5Acα2-3Galβ1-3GalNAcβ1-4(Neu5Acα2-3)Galβ1-4Glc-Sp21	7	6
174	GlcNAcα1-6Galβ1-4GlcNAcβ-Sp8	7	10
40	(4S)Galβ1-4GlcNAcβ-Sp8	7	8
385	Galβ1-4(Fucα1-3)GlcNAcβ1-6(Fucα1-4(Fucα1-2Galβ1-3)GlcNAcβ1-3)Galβ1-4Glc-Sp21	7	8
367	Neu5Acα2-6GlcNAcβ1-4GlcNAcβ1-4GlcNAc-Sp21	7	7
519	GalNAcα1-3(Fucα1-2)Galβ1-4GlcNAcβ1-6GalNAc-Sp14	7	5
97	GalNAcβ1-4(Fucα1-3)GlcNAcβ-Sp0	6	6
15	GalNAcβ-Sp8	6	12
265	Neu5Acα2-6GalNAcα-Sp8	6	8
137	Neu5Acb2-6(Galβ1-3)GalNAcα-Sp8	6	8
155	Galβ1-4(6S)Glcβ-Sp0	6	6
334	GalNAcβ1-3Galα1-4Galβ1-4GlcNAcβ1-3Galβ1-4Glcβ-Sp0	6	11
99	GalNAcβ1-4GlcNAcβ-Sp8	6	3

293	Galβ1-4GlcNAcβ1-3Galβ1-3GlcNAcβ-Sp0	6	2
30	(3S)Galβ1-3GlcNAcβ-Sp0	6	7
156	Galβ1-4(6S)Glcβ-Sp8	6	4
340	GlcNAcα1-4Galβ1-3GlcNAcβ-Sp0	6	11
596	Neu5Acα2-3Galβ1-4GlcNAcβ1-3Galβ1-4GlcNAcβ1-6(Neu5Acα2-3Galβ1-4GlcNAcβ1-3Galβ1-4GlcNAcβ1-3)GalNAcα-Sp14	6	6
610	Galβ1-3GalNAcβ1-4(Neu5Acα2-8Neu5Acα2-8Neu5Acα2-3)Galβ1-4Glcβ-Sp21	6	5
444	(6S)Galβ1-3(6S)GlcNAc-Sp0	6	2
197	Glcα1-6Glcα1-6Glcβ-Sp8	6	3
235	Neu5Acα2-6(Neu5Acα2-3)GalNAcα-Sp8	6	4
306	GalNAcβ1-3Galβ-Sp8	6	2
409	Galα1-3(Fucα1-2)Galβ1-4(Fucα1-3)Glcβ-Sp21	6	3
242	Neu5Acα2-3Galβ1-3(6S)GalNAcα-Sp8	6	6
471	Neu5Acα2-3Galβ1-4GlcNAcβ1-6(Neu5Acα2-3Galβ1-4GlcNAcβ1-3)GalNAcα-Sp14	6	3
160	Galβ1-4GlcNAcβ1-3GalNAc-Sp14	6	4
69	Fucα1-2Galβ1-4(Fucα1-3)GlcNAcβ1-3Galβ1-4(Fucα1-3)GlcNAcβ-Sp0	6	2
163	Galβ1-4GlcNAcβ1-3Galβ1-4GlcNAcβ-Sp0	6	2
383	Galβ1-4(Fucα1-3)GlcNAcβ1-6(Galβ1-3GlcNAcβ1-3)Galβ1-4Glc-Sp21	6	6
49	Neu5,9Ac2α2-6Galβ1-4GlcNAcβ-Sp8	6	3
225	GalNAcβ1-4(Neu5Acα2-8Neu5Acα2-8Neu5Acα2-8Neu5Acα2-3)Galβ1-4Glcβ-Sp0	6	4
259	Neu5Acα2-3Galβ1-4GlcNAcβ-Sp0	6	6
154	Galβ1-4(Fucα1-3)GlcNAcβ1-3Galβ1-4(Fucα1-3)GlcNAcβ1-3Galβ1-4(Fucα1-3)GlcNAcβ-Sp0	6	3
77	Fucα1-2Galβ1-4Glcβ-Sp0	6	5
75	Fucα1-2Galβ1-4GlcNAcβ-Sp0	6	4
198	Glcβ1-4Glcβ-Sp8	5	4
276	Neu5Acα2-8Neu5Acα2-3Galβ1-4Glcβ-Sp0	5	3
142	Galβ1-3GalNAcβ-Sp8	5	3
28	(3S)Galβ1-3(Fucα1-4)GlcNAcβ-Sp8	5	3
337	Neu5Acα2-3Galβ1-4(Fucα1-3)GlcNAcβ1-6(Neu5Acα2-3Galβ1-3)GalNAc-Sp14	5	3
181	GlcNAcβ1-3Galβ-Sp8	5	19
243	Neu5Acα2-6(Neu5Acα2-3Galβ1-3)GalNAcα-Sp8	5	9
119	Galα1-4(Fucα1-2)Galβ1-4GlcNAcβ-Sp8	5	5
410	Galβ1-4GlcNAcβ1-6(Neu5Acα2-6Galβ1-3GlcNAcβ1-3)Galβ1-4Glc-Sp21	5	2
189	GlcNAcβ1-4GlcNAcβ1-4GlcNAcβ1-4GlcNAcβ1-4GlcNAcβ1-4GlcNAcβ1-Sp8	5	4
62	Fucα1-2Galβ1-3GalNAcα-Sp14	5	4

341	GlcNAcα1-4Galβ1-4GlcNAcβ1-3Galβ1-4Glcβ-Sp0	5	12
252	Neu5Acα2-3Galβ1-4(Fucα1-3)(6S)GlcNAcβ-Sp8	5	7
182	GlcNAcβ1-3Galβ1-4GlcNAcβ-Sp0	5	6
260	Neu5Acα2-3Galβ1-4GlcNAcβ-Sp8	5	2
461	Neu5Acα2-3Galβ1-4GlcNAcβ1-6(Neu5Acα2-3Galβ1-4GlcNAcβ1-2)Manα1-6(GlcNAcβ1-4)(Neu5Acα2-3Galβ1-4GlcNAcβ1-2Manα1-3)Manβ1-4GlcNAcβ1-4GlcNAcβ-Sp21	5	8
486	Neu5Acα2-3Galβ1-3GlcNAcβ1-2Manα1-6(GlcNAcβ1-4)(Neu5Acα2-3Galβ1-3GlcNAcβ1-2Manα1-3)Manβ1-4GlcNAcβ1-4GlcNAc-Sp21	5	4
268	Neu5Acα2-6Galβ1-4GlcNAcβ-Sp0	5	5
563	GalNAcβ1-4GlcNAcβ1-3GalNAcβ1-4GlcNAcβ-Sp0	5	5
553	Neu5Acα2-8Neu5Gcα2-3Galβ1-4GlcNAc-Sp0	5	7
85	GalNAcα1-3(Fucα1-2)Galβ1-4GlcNAcβ-Sp0	5	3
105	Galα1-3(Fucα1-2)Galβ1-4GlcNAc-Sp0	5	8
178	GlcNAcβ1-6(GlcNAcβ1-3)Galβ1-4GlcNAcβ-Sp8	5	5
76	Fucα1-2Galβ1-4GlcNAcβ-Sp8	5	3
357	KDNα2-6Galβ1-4GlcNAc-Sp0	4	9
267	Neu5Acα2-6Galβ1-4(6S)GlcNAcβ-Sp8	4	6
499	Fucα1-2Galβ1-4GlcNAcβ1-2Manα-Sp0	4	5
162	Galβ1-4GlcNAcβ1-3Galβ1-4GlcNAcβ1-3Galβ1-4GlcNAcβ-Sp0	4	4
479	Neu5Acα2-3Galβ1-4GlcNAcβ1-6GalNAcα-Sp14	4	7
98	GalNAcβ1-4GlcNAcβ-Sp0	4	4
555	Neu5Gcα2-8Neu5Gcα2-3Galβ1-4GlcNAcβ1-3Galβ1-4GlcNAc-Sp0	4	2
107	Galα1-3(Fucα1-2)Galβ-Sp8	4	5
273	Neu5Acα2-6Galβ1-4Glcβ-Sp8	4	3
502	Neu5Acα2-6GalNAcβ1-4(6S)GlcNAcβ-Sp8	4	4
320	GlcNAcβ1-2Manα1-6(Neu5Acα2-6Galβ1-4GlcNAcβ1-2Manα1-3)Manβ1-4GlcNAcβ1-4GlcNAcβ-Sp12	4	1
13	Glcβ-Sp8	4	10
146	Galβ1-3Galβ-Sp8	3	2
302	Galβ1-4GlcNAcβ1-6(Galβ1-4GlcNAcβ1-3)Galβ1-4GlcNAc-Sp0	3	1
168	Galβ1-4GlcNAcβ-Sp0	3	5
521	Galα1-3Galβ1-4GlcNAcβ1-2Manα-Sp0	3	5
381	Galβ1-3GalNAcα1-3(Fucα1-2)Galβ1-4GlcNAc-Sp0	3	8
34	(3S)Galβ1-4(6S)GlcNAcβ-Sp0	3	6
78	Fucα1-2Galβ-Sp8	3	4
332	Neu5Acα2-6Galβ1-4GlcNAcβ1-3Galβ1-4GlcNAcβ1-3Galβ1-4GlcNAcβ-Sp0	3	2

44	(6S)Galβ1-4GlcNAcβ-Sp8	3	7
415	GalNAcα1-3GalNAcβ1-3Galα1-4Galβ1-4Glcβ-Sp0	3	4
434	GlcNAcβ1-6(GlcNAcβ1-2)Manα1-6(GlcNAcβ1-4)(GlcNAcβ1-4(GlcNAcβ1-2)Manα1-3)Manβ1-4GlcNAcβ1-4GlcNAc-Sp21	3	6
366	Neu5Acα2-6GlcNAcβ1-4GlcNAc-Sp21	3	4
339	GlcNAcα1-4Galβ1-4GlcNAcβ-Sp0	3	6
112	Galα1-3GalNAcβ-Sp8	3	9
64	Fucα1-2Galβ1-3GalNAcβ1-4(Neu5Acα2-3)Galβ1-4Glcβ-Sp9	3	5
169	Galβ1-4GlcNAcβ-Sp8	2	5
190	GlcNAcβ1-4GlcNAcβ1-4GlcNAcβ1-4GlcNAcβ1-4GlcNAcβ1-Sp8	2	2
312	MurNAcβ1-4GlcNAcβ-Sp10	2	2
101	Galα1-3(Fucα1-2)Galβ1-3GlcNAcβ-Sp0	2	8
506	GalNAcα1-3(Fucα1-2)Galβ1-3GlcNAcβ1-6GalNAcα-Sp14	2	6
224	Neu5Acα2-3Galβ1-3GalNAcα-Sp14	2	6
523	GalNAcα1-3(Fucα1-2)Galβ1-4 GlcNAcβ1-2Manα-Sp0	2	5
355	(6S)GlcNAcβ1-3Galβ1-4GlcNAcβ-Sp0	2	4
152	Galβ1-4(Fucα1-3)GlcNAcβ-Sp8	2	7
493	Galβ1-4(Fucα1-3)GlcNAcβ1-6(Neu5Acα2-6(Neu5Acα2-3Galβ1-3)GlcNAcβ1-3)Galβ1-4Glc-Sp21	2	7
256	Neu5Acα2-3Galβ1-4(Fucα1-3)GlcNAcβ1-3Galβ-Sp8	2	5
600	Neu5Acα2-3Galβ1-4GlcNAcβ1-3Galβ1-4GlcNAcβ1-6(Galβ1-3)GalNAcα-Sp14	2	5
393	GalNAcα1-3(Fucα1-2)Galβ1-3GalNAcα1-3(Fucα1-2)Galβ1-4GlcNAcβ-Sp0	2	5
126	Galβ1-3(Fucα1-4)GlcNAcβ1-3Galβ1-4(Fucα1-3)GlcNAcβ-Sp0	2	3
73	Fucα1-2Galβ1-4GlcNAcβ1-3Galβ1-4GlcNAcβ-Sp0	2	1
552	Neu5Gcα2-8Neu5Gcα2-3Galβ1-4GlcNAc-Sp0	2	5
171	Galβ1-4Glcβ-Sp0	2	2
569	(3S)GlcAβ1-3Galβ1-4GlcNAcβ1-3Galβ1-4Glc-Sp0	2	4
65	Fucα1-2Galβ1-3GlcNAcβ1-3Galβ1-4Glcβ-Sp8	1	1
186	GlcNAcβ1-4-MDPLys	1	3
598	GlcNAcβ1-3Galβ1-4GlcNAcβ1-3Galβ1-4GlcNAcβ1-3GalNAcα-Sp14	1	2
130	Fucα1-4(Galβ1-3)GlcNAcβ-Sp8	1	3
68	Fucα1-2Galβ1-3GlcNAcβ-Sp8	1	7
175	GlcNAcβ1-2Galβ1-3GalNAcα-Sp8	1	2
196	Glcα1-4Glcα-Sp8	1	2
269	Neu5Acα2-6Galβ1-4GlcNAcβ-Sp8	1	3
272	Neu5Acα2-6Galβ1-4Glcβ-Sp0	1	5

10	Neu5Acα-Sp11	1	2
95	GalNAcβ1-3(Fucα1-2)Galβ-Sp8	1	3
500	Fucα1-2Galβ1-3(6S)GlcNAcβ-Sp0	1	7
558	GlcNAcβ1-3Galβ1-4GlcNAcβ1-6(GlcNAcβ1-3Galβ1-4GlcNAcβ1-2)Manα1-6(GlcNAcβ1-3Galβ1-4GlcNAcβ1-2Man α1-3)Manβ1-4GlcNAcβ1-4GlcNAc-Sp24	1	9
4	GalNAcα-Sp8	1	3
207	Manα1-2Manα1-2Manα1-3Manα-Sp9	0	3
270	Neu5Acα2-6Galβ1-4GlcNAcβ1-3Galβ1-4(Fucα1-3)GlcNAcβ1-3Galβ1-4(Fucα1-3)GlcNAcβ-Sp0	0	6
507	GlcNAcβ1-6(GlcNAcβ1-2)Manα1-6(GlcNAcβ1-4)(GlcNAcβ1-4(GlcNAcβ1-2)Manα1-3)Manβ1-4GlcNAcβ1-4(Fucα1-6)GlcNAc-Sp21	0	5
245	Neu5Acα2-3Galβ-Sp8	0	3
441	Neu5Acα2-3Galβ1-4GlcNAcβ1-3Galβ-Sp8	0	2
557	Neu5Acα2-8Neu5Acα2-3Galβ1-4GlcNAc-Sp0	0	4
86	GalNAcα1-3(Fucα1-2)Galβ1-4GlcNAcβ-Sp8	0	3
84	(3S)Galβ1-4(Fucα1-3)Glcβ-Sp0	0	2
296	4S(3S)Galβ1-4GlcNAcβ-Sp0	-1	7
325	Neu5Acα2-3Galβ1-4GlcNAcβ1-2Manα1-6(Neu5Acα2-3Galβ1-4GlcNAcβ1-2Manα1-3)Manβ1-4GlcNAcβ1-4GlcNAcβ-Sp12	-1	3
604	GlcNAcβ1-6(Neu5Acα2-3Galβ1-3)GalNAcα-Sp14	-1	7
335	GalNAcα1-3(Fucα1-2)Galβ1-4GlcNAcβ1-3Galβ1-4GlcNAcβ-Sp0	-1	6
266	Neu5Acα2-6GalNAcβ1-4GlcNAcβ-Sp0	-1	3
173	GlcNAcα1-3Galβ1-4GlcNAcβ-Sp8	-2	2
166	Galβ1-4GlcNAcβ1-6(Galβ1-3)GalNAcα-Sp8	-2	5
294	Neu5Acα2-3Galβ1-3GlcNAcβ1-3Galβ1-3GlcNAcβ-Sp0	-2	6
129	Galβ1-3(Fucα1-4)GlcNAc-Sp8	-4	4
90	GalNAcα1-3(Fucα1-2)Galβ-Sp18	-4	2
164	Galβ1-4GlcNAcβ1-3Galβ1-4Glcβ-Sp0	-5	5
108	Galα1-3(Fucα1-2)Galβ-Sp18	-6	2
